# Network propagation-based prioritization of long tail genes in 17 cancer types

**DOI:** 10.1186/s13059-021-02504-x

**Published:** 2021-10-07

**Authors:** Hussein Mohsen, Vignesh Gunasekharan, Tao Qing, Montrell Seay, Yulia Surovtseva, Sahand Negahban, Zoltan Szallasi, Lajos Pusztai, Mark B. Gerstein

**Affiliations:** 1 Computational Biology & Bioinformatics Program, Yale University, New Haven, CT 06511 USA; 2 Breast Medical Oncology, Yale School of Medicine, New Haven, CT 06511 USA; 3 Yale Center for Molecular Discovery, Yale University, West Haven, CT 06516 USA; 4 Department of Statistics & Data Science, Yale University, New Haven, CT 06511 USA; 5 Children’s Hospital Informatics Program, Harvard-MIT Division of Health Sciences and Technology, Harvard Medical School, Boston, MA 02115 USA; 6 Department of Molecular Biophysics and Biochemistry, Yale University, New Haven, CT 06511 USA; 7 Department of Computer Science, Yale University, New Haven, CT 06511 USA

## Abstract

**Background:**

The diversity of genomic alterations in cancer poses challenges to fully understanding the etiologies of the disease. Recent interest in infrequent mutations, in genes that reside in the “long tail” of the mutational distribution, uncovered new genes with significant implications in cancer development. The study of cancer-relevant genes often requires integrative approaches pooling together multiple types of biological data. Network propagation methods demonstrate high efficacy in achieving this integration. Yet, the majority of these methods focus their assessment on detecting known cancer genes or identifying altered subnetworks. In this paper, we introduce a network propagation approach that entirely focuses on prioritizing long tail genes with potential functional impact on cancer development.

**Results:**

We identify sets of often overlooked, rarely to moderately mutated genes whose biological interactions significantly propel their mutation-frequency-based rank upwards during propagation in 17 cancer types. We call these sets “upward mobility genes” and hypothesize that their significant rank improvement indicates functional importance. We report new cancer-pathway associations based on upward mobility genes that are not previously identified using driver genes alone, validate their role in cancer cell survival in vitro using extensive genome-wide RNAi and CRISPR data repositories, and further conduct in vitro functional screenings resulting in the validation of 18 previously unreported genes.

**Conclusion:**

Our analysis extends the spectrum of cancer-relevant genes and identifies novel potential therapeutic targets.

**Supplementary Information:**

The online version contains supplementary material available at 10.1186/s13059-021-02504-x.

## Background

Rapid developments in sequencing technologies allowed comprehensive cataloguing of somatic mutations in cancer. Early mutation-frequency-based methods identified highly recurrent mutations in different cancer types, many of which were experimentally validated as functionally important in the transformation process and are commonly referred to as cancer driver mutations. However, the biological hypothesis that recurrent mutations in a few driver genes account fully for malignant transformation turned out to be overly simplistic. Recent studies indicate that some cancers do not harbor any known cancer driver mutations, and all cancers carry a large number of rarely recurrent mutations in unique combinations in hundreds of potentially cancer-relevant genes [1–7]. These genes are part of a long tail in mutation frequency distributions and referred to as “long tail” genes.

Many long tail mutations demonstrated functional importance in laboratory experiments, but studying them all and assessing their combined impact is a daunting task for experimentalists. This creates a need for new ways to estimate the functional importance and to prioritize long tail mutations for functional studies. A central theme in finding new associations between genes and diseases relies on the integration of multiple data types derived from gene expression analysis, transcription factor binding, chromatin conformation, or genome sequencing and mechanistic laboratory experiments. Protein-protein interaction (PPI) networks are comprehensive and readily available repositories of biological data that capture interactions between gene products and can be useful to identify novel gene-disease associations or to prioritize genes for functional studies. In this paper, we rely on a framework that iteratively propagates information signals (i.e., mutation scores or other quantitative metrics) between each network node (i.e., gene product) and its neighbors.

Propagation methods have often leveraged information from genomic variation, biological interactions derived from functional experiments, and pathway associations derived from the biomedical literature. Studies consistently demonstrate the effectiveness of this type of methods in uncovering new gene-disease and gene-drug associations using different network and score types. Nitsch et al. [8] is one of the early examples that used differential expression-based scores to suggest genes implicated in disease phenotypes of transgenic mice. A study by Lee et al. shortly followed to suggest candidate genes using similar propagation algorithms in Crohn’s disease and type 2 diabetes [9]. Other early works that use propagation account for network properties such as degree distributions [10] and topological similarity between genes [11–13] to predict protein function or to suggest new candidate genes.

Cancer has been the focus of numerous network propagation studies. We divide these studies into two broad categories: (A) methods that initially introduced network propagation into the study of cancer, often requiring several data types, and (B) recent methods that utilize genomic variation, often focusing on patient stratification and gene module detection (for a complete list, see [14]).

Köhler et al. [15] used random walks and diffusion kernels to highlight the efficacy of propagation in suggesting gene-disease associations in multiple disease families including cancer. The authors made comprehensive suggestions and had to choose a relatively low threshold (0.4) for edge quality filtering to retain a large number of edges given the limitations in PPI data availability in 2008. Shortly afterwards, Vanunu et al. [16] introduced PRINCE, a propagation approach that leverages disease similarity information, known disease-gene associations, and PPI networks to infer relationships between complex traits (including prostate cancer) and genes. Propagation-based studies in cancer rapidly cascaded to connect gene sequence variations to gene expression changes using multiple diffusions [17]; to generate features used to train machine learning models that predict gene-disease associations in breast cancer, glioblastoma multiforme, and other cancer types [18, 19]; or to suggest drug targets in acute myeloid leukemia by estimating gene knockout effects in silico [20].

Hofree et al. introduced network-based stratification (NBS) [21], an approach that runs propagation over a PPI network to smoothen somatic mutation signals in a cohort of patients before clustering samples into subtypes using non-negative matrix factorization. Hierarchical HotNet [22] is another approach that detects significantly altered subnetworks in PPI networks. It utilizes propagation and scores derived from somatic mutation profiles as its first step to build a similarity matrix between network nodes, constructs a threshold-based hierarchy of strongly connected components, then selects the most significant hierarchy cutoff according to which mutated subnetworks are returned. Hierarchical HotNet makes better gene selections than its counterparts with respect to simultaneously considering known and candidate cancer genes, and it builds on two earlier versions of HotNet (HotNet [23] and HotNet2 [24]).

These studies have addressed varying biological questions towards a better understanding of cancer, and they have faced limitations with respect to (i) relying on multiple data types that might not be readily available [17, 18], (ii) limited scope of biological analysis that often focused on a single cancer type [17, 20], (iii) suggesting too many [20] or too few [19] candidate genes, or (iv) being focused on finding connected subnetworks, which despite its demonstrated strength as an approach to study cancer at a systems level might miss lone players or understudied genes [17, 22–24]. To address these issues and parallel the emerging focus on long tail genes and non-driver mutations [2, 4, 5, 25–29], we build on the well-established rigor of propagation and introduce a new approach that particularly prioritizes rarely to moderately mutated genes implicated in cancer. Our analysis spans 17 cancer types and relies centrally on two data types: mutation frequency and PPI connectivity data. We hypothesize that a subset of long tail genes, originally with low mutation frequency ranks, can leverage their positionality in PPI networks and the mutational burden within their extended neighborhoods to play an important role in cancer as signaled by the much higher individual ranks they attain after propagation. These genes are not merely pinpointed based on their high post-propagation ranks, but rather on the strong improvement in their pre- and post-propagation ranking difference that exceeds stringent measures. Hence, we describe these genes throughout this paper as upward mobility genes (UMGs). To the limits of our knowledge, this is the first propagation approach that focuses entirely on long tail genes.

We efficiently identify a considerable number of UMGs (*n* = 28–83 per cancer type) and demonstrate their functional importance in cancer on multiple levels. Using somatic mutation data from the TCGA and two comprehensive PPI networks with significant topological differences, STRING v11 and HumanNet v2, we detect UMGs in BRCA, CESC, CHOL, COAD, ESCA, HNSC, KICH, KIRC, KIRP, LIHC, LUAD, LUSC, PRAD, READ, STAD, THCA, and UCEC. These genes reveal a significant number of regulatory pathway associations that would be overlooked when relying on known driver genes alone. Furthermore, in silico analysis demonstrates that UMGs exert a highly significant effect on cancer cell survival in vitro with cancer type specificity, and they outperform genes suggested by other network methods with respect to this impact on cancer cell survival. We then validate a previously unreported subset of the identified genes in vitro through siRNA knockout experiments. Finally, we perform an analysis of UMGs’ positionality in a combined STRING-HumanNet v2 PPI network to classify each UMG as a potential cancer driver, drug target, or both. Together with known drivers, we hope that UMGs will draw a more complete portrait of the disease.

## Results

### Overview

**Fig. 1:**
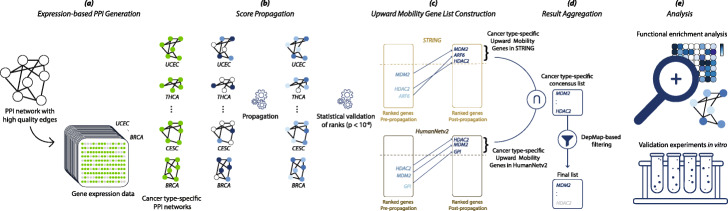
Schematic overview of the UMG identification strategy

First, we generate PPI networks specific to each of the 17 cancer types in the TCGA using only genes that are expressed in a given cancer type (Fig. 1a and Additional file 1: Table S1). We use the STRING and HumanNet v2 networks that have different topologies and information channels for constructing the networks and use only high-quality edges. We then perform propagation over each network, where each sample’s somatic mutation profile includes a quantized positive value ∈ [1, 4] for genes with mutations, and 0 otherwise (Fig. 1b). Next, we perform the Mann-Whitney *U* test to assess the significance of propagation-based rankings by measuring the enrichment of known functionally important COSMIC genes towards higher ranks in post-propagation lists. Results demonstrate high statistical significance across all studied cohorts (*p* < 10^−5^, Additional file 2: Table S2) demonstrating the validity of the method to identify genes with functional importance. We then calculate the difference in pre- (i.e., raw mutation frequency) and post-propagation ranking for each gene. Genes that move up in the rank order in the post-propagation list are called UMGs. We construct a preliminary UMG list for each cancer cohort based on the stringent final rank cutoff and upward rank increase (i.e., upward mobility) threshold. In this paper, genes whose rank significantly improves during propagation and land in a pre-defined top block of post-propagation ranked lists are retained (Fig. 1c). Using this strategy, our approach focuses on long tail genes and excludes frequently mutated genes (including classical cancer drivers) that occupy high ranks before propagation and therefore cannot meet the upward mobility threshold. We identify UMGs separately for each of the 17 cancer types. To further filter UMGs for potential functional importance, we remove genes with minimal or no impact on corresponding cancer cell survival after gene knockdown in the Cancer Dependency Map Project (DepMap) [30]. This step eliminates 4–13% of UMGs (Fig. 1d). We finally analyze the biological and topological properties of the shortlisted UMGs on pan-cancer and cancer type levels (Fig. 1e).

### UMGs across 17 cancer types

**Fig. 2:**
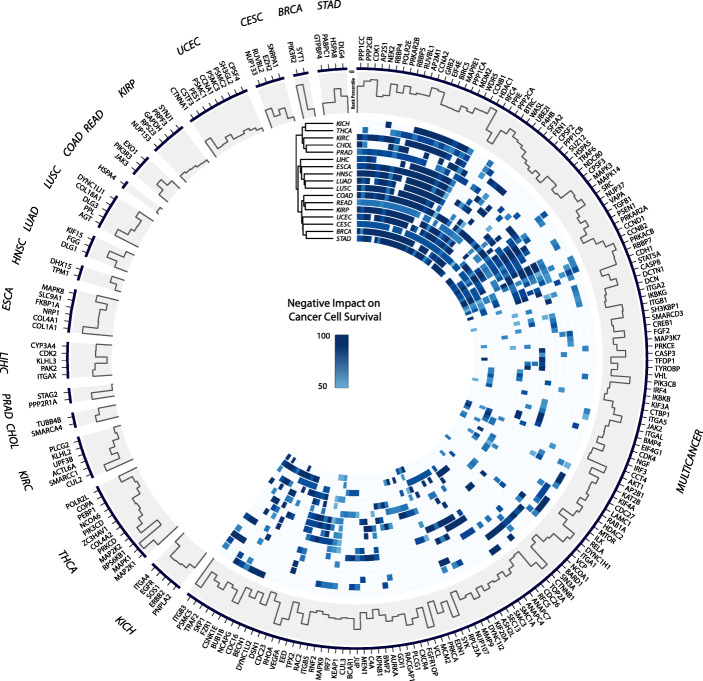
Distribution of UMGs across 17 cancer types. Right: genes in 2 or more cancer types. The dendrogram is based on the hierarchical clustering of heatmap rows. Each heatmap value corresponds to a percentage-based score of a cancer type’s cell lines whose survival is negatively impacted by a gene’s knockout. For each value, the maximum percentage across RNAi and CRISPR experiments is selected. Left: cancer type-specific genes. The histogram throughout the plot corresponds to the normalized rank of each UMG in the lists it belongs to

We report 230 UMGs across 17 cancer types. UMG lists capture the expected biological heterogeneity of cancer types: 76 genes (33%) are specific to one cancer type, 116 (50.4%) to 2–9 types, and only 38 (16.5%) to 10 or more types (Additional file 3: Table S3). The longest list of UMGs corresponds to CESC (*n* = 83 genes) and the shortest to CHOL (*n* = 28). Hierarchical complete linkage clustering of cancer types (right of Fig. 2) using UMG list membership and DepMap dependency scores of the genes (which reflect their importance in cell growth) reveals interesting patterns. Similar to results based on driver gene sets identified in [7], subsets of squamous (ESCA, HNSC, and LUSC) and gynecological (BRCA, CESC, and UCEC) cancers cluster together. Close clustering results also correspond to the lung (LUAD and LUSC) and colon and rectum (COAD and READ) as tissues of origin, while others match with the rates of driver mutations across cancer types (i.e., Fig. 1d in [7]), particularly (i) STAD and CESC; (ii) KIRP, READ, and COAD; and (iii) LUSC, LUAD, HNSC, ESCA, and LIHC, suggesting similarities between driver and long tail mutational patterns. Interestingly, UMGs specific to a single cancer type (left of Fig. 2) include a considerable number of genes whose products have similar functions such as *COL4A1* and *COL1A1* that encode different types of collagen (specific to ESCA), and triplets of genes that encode proteins in the 26S proteasome complex (*PSMC1/2/3*, specific to UCEC) and mitogen-activated kinases (*MAPK1* and *MAP2K1/2*, specific to THCA). Functional gene clusters shared among cancer types include *DYNC1LI2/I2/H1* that encode different components of the cytoplasmic dynein 1 complex and *PPP1CC/1CA/2CB/2CA* that encode subunits of protein phosphatase enzymes. The circos plot [31] of Fig. 2 shows the distribution of UMGs across cancer types, their relative ranks within UMG lists, and their impact on cancer type-specific cell survival.

### UMGs reveal known and novel cancer-pathway associations

**Fig. 3:**
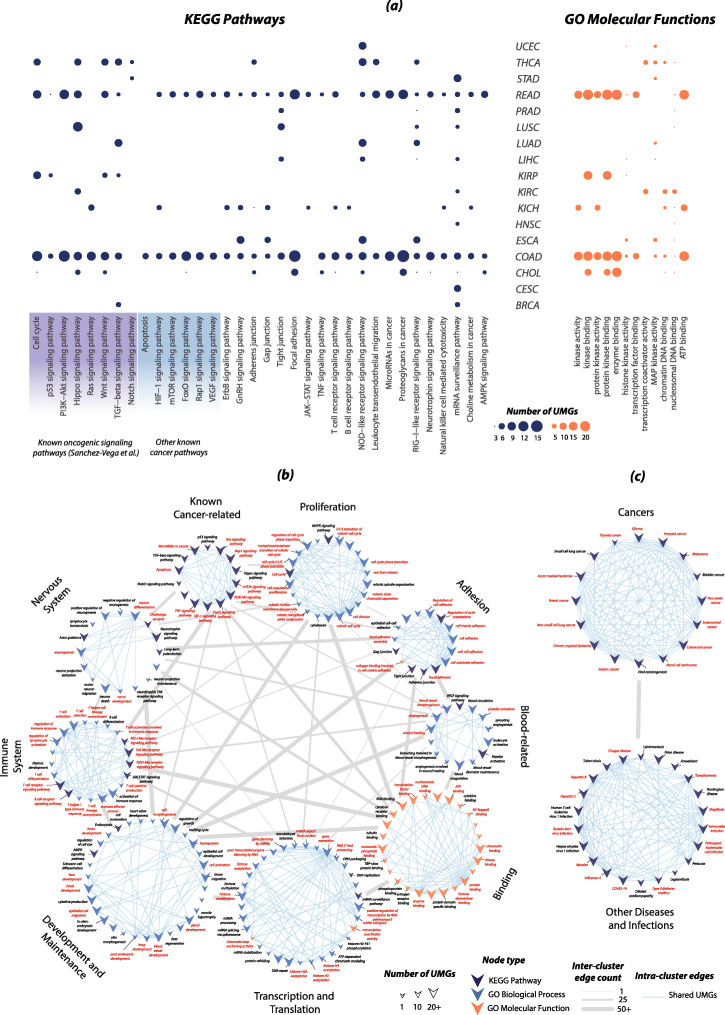
Biological enrichment results for UMGs at cancer type and pan-cancer levels. **a** UMGs uncover known and novel associations between cancer types and biological pathways. Enrichment analyses are performed for each cancer type’s combined list of UMGs and drivers. Shown results correspond to significant pathway and molecular function associations exclusively uncovered by UMGs. **b** Pan-cancer analysis of all 230 UMGs allows for the identification of biological pathways, processes, and functions strongly associated with UMGs (in red) that suggests potential therapeutic targets. **c** A similar analysis to **b** on clusters of KEGG mega-pathways uncover disease-disease and disease-infection associations pertaining to UMGs

Biological enrichment analysis of UMGs, separately and in combination with known drivers, confirms some already known functional importance of the UMGs and suggests new associations between cancer types and biological pathway alterations. UMGs analyzed alone or together with known cancer drivers have statistically significant associations (Benjamini *p-adjusted* < 0.05) with most of the oncogenic pathways (8 out of 10) curated by Sanchez-Vega et al. [32] (Fig. 3a). These results indicate that UMGs are members of known biological pathways and can broaden the study of biological processes that contribute to malignant transformation. This is particularly relevant in cancers where driver gene-based pathway associations revealed only a few relevant pathways (e.g., KICH and CHOL in [7]). Interestingly, the p53 pathway has only a small number of associations with UMGs in contrast to the many associations we detected with the TGF-beta and Hippo signaling pathways. Other known cancer pathways are also altered by UMGs and include Notch, HIF-1, and mTOR. Notably, the number of cancer type-specific pathway associations does not correlate with the size of UMG lists. For example, KICH, which has one of the smallest lists of UMGs (*n* = 41 genes), has a sizeable set of pathway associations, while CESC with the largest UMG list (*n* = 83) has considerably fewer associations. These findings suggest greater diversity in altered biological processes that lead to the development of KICH compared to CESC.

On the pan-cancer level, we partitioned enrichment results for all 230 UMGs into 9 major functional clusters based on biological function (Fig. 3b). Using EnrichmentMap (EM) [33], we built a network of intra- and inter-cluster similarity measured through gene overlap between enrichment entities (i.e., pathways, biological processes, and molecular functions; the “Methods” section). Connectivity patterns within the EM network provide insights into the sets of entities and UMGs. Within the clusters, we identified biological entities with high connectivity (red labels, Fig. 3b). These entities include oncogenic pathways, such as PI3K-AKT, RAS, and mTOR, and important biological processes including cell matrix adhesion and chromatin remodeling. Their high connectivity is often driven by a selected subset of UMGs with high frequency in their constituent edges (Table 1). Subsets of these frequent UMGs encode subunits of proteins and members of protein complexes with a strong association with cancer (e.g., *PIK3R2/R3/CB/CD*’s products in phosphatidylinositol kinases (PI3Ks) [34], and *IKBKB/G*’s products that are regulatory subunits in an inhibitor of the Nuclear Factor Kappa B kinase (NFKB) [35]). Given their significant and wide range of biological functionality, these genes constitute a potential subset of potent drug targets. A similar analysis on KEGG mega-pathways corresponding to diseases and infections revealed another subset of frequent UMGs and demonstrated that UMGs are generally important genes that participate in broader biological processes than cancer alone (Fig. 3c, Table 1). Observed associations include well-studied ones between multiple cancers and hepatitis C [36], type II diabetes mellitus [37, 38], and HTLV-I infection [39], and new ones such as the potential association with COVID-19 [40].

**Table 1:** Frequent UMGs driving high connectivity within EnrichmentMap functional clusters

Functional cluster	Frequent UMGs
Known cancer-related	*PIK3R2*, *PIK3R3*, *AKT1*, *IKBKB*, *MAPK1*, *MAPK3*, *PIK3CB*, *PIK3CD*, *MAP2K1*, *MAP2K2*
Proliferation	*CCND1*, *BUB1B*, *CDC16*, *ANAPC4*, *ANAPC7*, *CDC23*, *CDC26*, *CDC27*, *CUL3*, *TGFB1*, *AURKA*, *CDK1*, *CDK2*, *CDK4*, *CCNB1*, *NDC80*
Adhesion	*ITGB1*, *ITGB5*, *RHOA*, *SRC*, *ITGA2*, *ITGA4*, *VCL*
Transcription and translation	*RUVBL2*
Binding	*SRC*, *RELA*
Immune system	*TRAF6*, *MTOR*, *IRF4*, *IKBKB*, *IKBKG*
Cancer mega-pathways	*CCND1*, *PIK3R2*, *PIK3R3*, *GRB2*, *EGFR*, *AKT1*, *MAPK1*, *MAPK3*, *PIK3CB*, *SOS1*, *PIK3CD*, *MAP2K1*, *MAP2K2*
Other diseases and infection mega-pathways	*CASP3*, *MAPK14*, *CASP8*, *PIK3R2*, *PIK3R3*, *TRAF6*, *AKT1*, *MAP3K7*, *IRF3*, *IKBKB*, *IKBKG*, *MAPK1*, *MAPK3*, *PIK3CB*, *RELA*, *MAPK8*, *PIK3CD*, *MAPK9*

### UMGs impact survival of cancer cells in vitro

To assess the functional importance of UMGs in cancer cell survival in vitro, we obtained their cancer type-specific dependency scores from the DepMap project. DepMap reports results on the comprehensive genome-wide loss of function screening for all known human genes using RNA interference (RNAi) and CRISPR to estimate tumor cell viability after gene silencing in hundreds of cancer cell lines. The CRISPR dataset includes 990 cell lines, and the RNAi dataset includes 712 cell lines [30]. A dependency score of 0 corresponds to no effect on cell viability, and a negative score corresponds to impaired cell viability after knocking down the gene; the more negative the dependency score, the more important the gene is for cell viability. We used the most recent data release that accounts for batch and off-target effects and therefore provides more accurate estimates of functional impact [41].

We found that cancer type-specific mean dependency scores of UMGs are higher (i.e., more negative) than non-UMGs’ across all 17 cancer types, and in both CRISPR and RNAi experiments. This indicates that knockout of UMGs consistently yields a stronger negative effect on cancer cell survival than that of non-UMGs (Mann-Whitney *U* test, *p* < 5 × 10^−3^, Additional file 4: Table S4, the “Methods” section).

Our UMG detection method is entirely focused on prioritizing long tail genes for functional importance. Most existing network methods focus their assessment on uncovering known cancer genes or are geared towards other goals—such as detecting subnetworks that maximize coverage of mutational profiles or are highly mutated—and therefore may be less efficient to prioritize long tail genes. To have a better understanding of the specifications of UMGs, we compared their impact on the survival of cancer cell lines to that of non-driver genes selected by five other methods. Three of these methods are propagation-based and include FDRNet [42], Hierarchical HotNet (HHotNet) [22]—in 3 different settings, and Zhou et al.’s propagation algorithm that resembles random walk with restart—in its original and edge-normalized settings [43]. The other two include nCOP [44], a non-propagation network method that recently demonstrated an ability to uncover non-driver genes across multiple cancer types, and MutSig [45], which identifies genes mutated more often than expected in a given cohort. HHotNet reported statistically significant results after the integration over both PPI networks in only 5 out of the 17 cancer types. Hence, we included two other settings (largest and all subnetworks) where the method was able to report statistically significant results in one network. FDRNet successfully generated results on STRING, and its reported results across cancer types are based on this network (the “Methods” section).

**Fig. 4:**
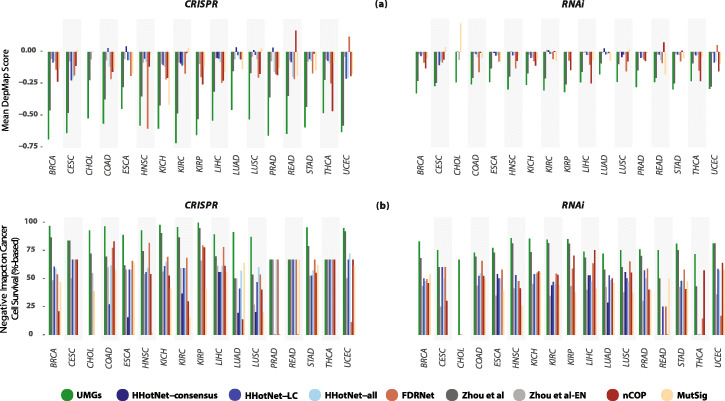
Comparisons with other methods. **a** UMGs demonstrate a considerably stronger (CRISPR- and RNAi-measured) impact on survival of cancer cell lines than other non-driver genes suggested by HHotNet (in 3 settings), FDRNet, Zhou et al. (in its original and edge-normalized settings), nCOP, and MutSig. Higher negative values indicate a greater negative effect on cell survival after gene knockdown. **b** UMGs’ strong impact on the survival of cancer cell lines is significantly broader than that of genes selected by other methods. The median percentage-based score of cancer cell lines negatively impacted by UMGs’ knockout is consistently higher with cancer type specificity

Almost all methods’ generated gene sets had a knockdown negative impact on cancer cell survival, but UMGs had the strongest impact across cancer types and in both CRISPR and RNAi experiments (Fig. 4a). The median percentage-based score of cell lines negatively impacted by UMGs’ knockout is also consistently higher than that for genes selected by the other methods in 28 out of the 34 cancer type-assay combinations (Fig. 4b), with the remaining 6 including 4 ties. Notably, a number of UMGs have an extremely strong negative impact on cell survival across cancer types. For instance, PRAD, READ, and THCA sets include genes with mean DepMap CRISPR score < −2 in their cell lines, and all other cancer types except HNSC include genes with score < −1.7. Similar results were also obtained for these comparisons before the optional DepMap filtering step that only removed 4–13% of UMGs (Additional file 5: Fig. S1). As FDRNet, HHotNet, Zhou et al*.*, nCOP, and MutSig do not solely focus on long tail genes and gene sets generated by these methods include known cancer drivers, we performed the same comparisons after including known cancer-specific drivers from all gene lists, which also produced similar results (Additional file 5: Fig. S2). Concurrently including both subsets of UMGs (pre-DepMap filtering and drivers) produced similar results across cancer types as well (Additional file 5: Fig. S3).

### UMGs as “weak drivers” and potential novel drug targets

The aim behind identifying UMGs is to expand the repertoire of cancer-relevant genes in line with recent studies whose results defy the neutrality of long tail genes or passenger mutations in carcinogenesis [2, 4, 5, 25–29]. In this section, we categorize each UMG as a potential “weak driver” that may complement known drivers, a candidate drug target whose inhibition could arrest cancer growth, or both, based on positionality in PPI networks relative to currently known drivers.

**Fig. 5:**
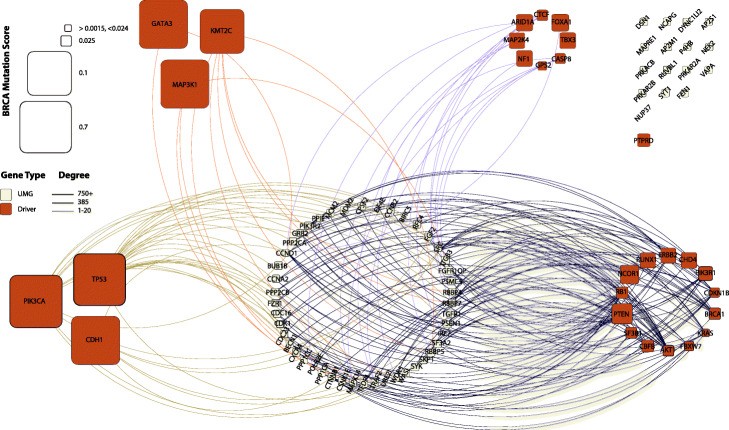
PPI network analysis of the relationships between UMGs (white nodes) and known driver genes (red) in breast invasive carcinoma (BRCA) suggests roles of UMGs. Driver genes are split into categories based on initial mutation score and node degree: (i) high score, high degree (bottom left); (ii) high score, low degree (top left); (iii) low score, low degree (top right); and (iv) low score, high degree (bottom right). UMGs connected to driver subsets (i) and (ii) (olive and orange edges) and ones with no mutation score (e.g., *POLR2E*) are likely to be drug targets. UMGs connected to (iii) and (iv) and ones without connections to drivers (top right corner, e.g., *DSN1*) are likely to be “weak drivers.” Other cancer types’ results are available in Additional file 5: Figs. S4-19

In the propagation framework we use, two of the most important factors that determine a node’s score after convergence are the number of high scoring nodes within its neighborhood and the connectivity of these neighbors. For a node to rank higher, the best case scenario involves having near exclusive connections with multiple neighbors (*k* ≥ 1 steps) whose initial scores are high. We examine these properties of each cancer type’s UMGs. We use a composite PPI network that merges signals from STRING and HumanNet v2 by including the union of high-quality edges of both networks. Figure 5 shows a representative network that corresponds to BRCA, with all others included in the supplement (Additional file 5: Figs. S4-19). For convenience in visualization, we include immediate neighborhoods of each node and the UMG-driver edges only.

The first category of UMGs includes genes connected to high scoring known drivers (Fig. 5 left side, olive and orange edges). By virtue of sharing connections with these frequently mutated drivers, this subset of UMGs likely includes cancer type-specific potential drug targets. The most promising UMG drug target candidates are those connected to high degree, high scoring drivers (via olive edges). Building on the same reasoning, low scoring drivers might not be the dominating force driving cancer across the majority of samples. UMGs connected to these low scoring drivers (Fig. 5 right side, dark blue and purple edges) constitute the second category and are considered potential supplementary drivers that enhance the driver function. The third category includes UMGs with nearly no observed mutations in the TCGA cohort (i.e., very low initial score). These UMGs often form a small subset and are likely to be drug targets or false positives limited by the size of the cohort under study. In Fig. 5 (and Additional file 5: Figs. S4-19), they can be distinguished by their lack of node border (e.g., 6 genes in Fig. 5: *NUP37*, *UBE21*, *POLR2E*, *IRF7*, *BIRC5*, and *EIF4E*). The fourth category includes UMGs with positive initial score and no connections to driver genes (Fig. 5, top right grid). These genes’ positive scores and connectivity with non-drivers significantly lift their rank during propagation and render them potentially overlooked weak drivers. While most UMGs are designated either potential drug targets or weak drivers, others are connected to multiple types of driver genes and accordingly might be considered for both (e.g., *RBBP5* with multi-colored edges in Fig. 5). We also point out the well-connectedness of many UMGs, which in part allows them to have enough upward mobility to be detected by our approach. Yet, UMGs tend to have a considerably smaller number of neighbors compared to very well-studied drivers such as *TP53*, *PIK3CA*, and *BRCA1*.

### UMGs bridge gaps in the literature and suggest novel genes

The study of cancer has long been interdisciplinary, often in the realms of various scientific and medical spheres. Disciplinary paradigms evolved over time to produce varying types of associations between genes and cancers. To further estimate the functional importance of UMGs, we manually cross-referenced our UMG lists with publications and found that a large percentage of UMGs have been previously reported to play a role in cancer based on functional experiments. This percentage is as high as 85% of UMGs in cancer types like BRCA. Surprisingly, the same percentage drops to only 31% when we used CancerMine to find literature-based associations. CancerMine is an automated tool that applies text mining on existing literature to report drivers, oncogenes, or tumor suppressors across cancers. Similar results were obtained across cancer types (Additional file 6: Table S5).

### Screening experiments validate 18 new genes in vitro

We performed a series of siRNA knockdown experiments in vitro to validate the DepMap results and to confirm the functional importance of selected UMGs. We selected 29 UMGs that have not been reported in the literature to be tested in gene knockdown experiments in the context of any cancer phenotype (the “Methods” section). We used 7 cell lines representing 3 types of cancer, namely H460 and HCC1299 from the lung; MDAMB231, MDAMB468, BT549, and HCC187 from the breast; and DU145 from prostate cancer.

Experimental results further underscore the efficacy of UMG detection to uncover functionally important long tail genes. The knockdown of 18 out of these 29 UMGs (62%) significantly decreased cell survival in 1 to 5 cell lines exceeding the threshold of 3 standard deviations with respect to negative control samples (the “Methods” section, Additional file 7: Table S6). We note that several UMGs demonstrated cell line specificity while others had a more widespread effect (affects 5/7 cell lines). These newly cancer-relevant genes have already known functions in regulating immune response (*AP2M1*, *DCTN1*, *CCT4*, *DYNC1I2*, and *DYNC1LI2*), kinase binding (*DLG3*), cell cycle progression (*SEC13*, *ANAPC7*, *CDC26*, *PSMC3*, *PPP1CC*), DNA repair (*PPIE*, *RFC5*, *POLR2E*, and *POLR2L*), cell death (*VAPA*), and mRNA splicing (*SF3A2*). The list also includes *PNPLA2*, which encodes for an enzyme associated with transacetylase activity.

## Discussion

Biological analysis of UMGs demonstrates strong correlations with studies performed on known cancer drivers. It also unlocks a wide range of potential associations between key pathways and cancer types and allows for classifying UMGs based on their centrality to biological functions, which in turn opens the door for a more informed drug targetability. Based on their network positionality, we propose that UMGs include “weak drivers” and cancer type-specific drug targets. Manual curation of the literature confirmed that many of our UMGs were previously implicated in cancer biology in various ways, but we also identified previously unstudied potential cancer-relevant genes. Yet, results suggest that we have not reached a point of data saturation with respect to analyzing long tail genes. The generation of new and larger datasets will likely improve UMG prioritization for rare cancer types such as cholangiocarcinoma (CHOL) and chromophobe renal cell carcinoma (KICH). As the functional importance and centrality of known and new cancer-relevant genes changes, network propagation results and UMG rankings will likely follow suit. This was already evident in our PPI positionality analysis: with 3 or less known genes identified in KICH and READ in Bailey et al. [7] and COSMIC [46], respectively, most of these cancer types’ UMGs belong to the third and fourth categories (near-zero mutation scores and no connections with drivers, Additional file 5: Figs. S9 and S16). Another example is CHOL, with its small cohort that brings most UMGs into the third category (no observed mutations, Additional file 5: Fig. S5).

In their current arrangement in the circos plot of Fig. 2, we also posit that the confidence associated with UMGs increases in a roughly clockwise direction, with the highest confidence to be associated with cancer type-specific genes. The incorporation of additional functional genomics data (e.g., noncoding mutations and methylation data), coupled with improvements in the accuracy of reported PPIs, will strengthen our knowledge on the role of UMGs and long tail genes more broadly. Finally, we note that bridging gaps across disciplines is often essential to biomedical knowledge production. The oncogenic validation of potential drug targets in UMGs also remains central to changing their status from potential to clinically actionable ones.

## Conclusions

In this paper, we describe a new network propagation-based approach that is particularly well suited to estimate the functional importance of rarely mutated long tail genes in cancer. The method is computationally efficient and is based on change in ranking before versus after network propagation. We show that upward mobility genes that attain significant improvements in mutation score-based ranking after propagating through PPI networks are enriched in functionally relevant genes. By virtue of high post-propagation ranks, cancer-related biological function, and significantly strong impact on cancer cell line survival, our approach prioritizes long tail genes across 17 cancer types. To reduce false positivity rate, we integrate results over two major PPI networks, filter out nodes whose genes are unexpressed in each cancer type’s tumor samples, and statistically validate rankings and cell survival impact. Computational and in vitro analyses further highlight the importance of reported genes and open the door for an expanded spectrum of gene cancer relevance.

## Methods

### Mutation matrix generation

Variants from the TCGA MC3 somatic mutation dataset (*n* = 3.6 M) are used to generate initial scores for each of the 17 cancer types. A sample-gene matrix for each cancer type includes mutation counts restricted to splicing and coding exonic variants. Counts are then normalized by gene length, and each resulting non-zero value is finally converted to a discrete number in {1, 2, 3, 4} based on its position with respect to 50th, 70th, and 90th percentiles in the cancer type-specific normalized mutation frequency distribution. Gene ranks before and after propagation are calculated based on the mean frequency within each cohort.

### PPI network processing

We adopt the broad definition of protein-protein interactions that encompasses direct physical interactions alongside indirect functional ones derived from co-expression, gene fusion, text mining, co-essentiality, and pathway membership datasets among others. We perform edge filtering on both PPI networks and retain edges with a confidence score equal to or higher than 0.7 across all information channels in STRING v11 and the top 10% of edges in HumanNet v2. The networks after this filtering have |*V*| = 17,130 and 11,360 vertices and |*E*| = 419,772 and 37,150 undirected edges, respectively. We then generate cancer type-specific PPI networks by selecting the largest connected component in each network and filtering out (proteins of) genes unexpressed in the tumor samples of each cancer type (i.e., genes with FPKM > 15 in > 20% of tumor samples are retained). The sizes of the resulting PPI networks are different and listed by cancer type in Additional file 1: Table S1.

### Propagation score calculation

To calculate propagation scores, we use an approach that imitates random walk with restart [43]. Briefly, let the PPI network be represented as *G* = (*V*, *E*), where *V* is the set of gene products and *E* is the set of edges. Furthermore, let *W* be the weighted adjacency matrix of *G*. We choose to normalize *W* such that *W’* = *W* . *D*^*−1*^, where *D* is the diagonal matrix of columns sums in $W:D=\mathit{\operatorname{diag}}\left({\sum}_{i=1}^{\left|G\right|}{W}_{ij}\right),j\in \left\{1,2,\dots, |G|\right\}.$

Let *M* be a |*G*| × *N* matrix with somatic mutation profiles of *N ≥* 1 samples over genes from which *G*’s nodes originate before transcription. *S*_*ij*_ is a positive value for each *g*_*i*_ ∈ *G* with mutations in sample s_*j*_ ∈ *S*, and 0 otherwise. Propagation is then executed within each sample until convergence according to the following function:

$$ {S}^{\left(t+1\right)}=\alpha W\hbox{'}{S}^{(t)}+\left(1-\alpha \right){S}^{(0)} $$

where *S*^*(0)*^ *= M* and *α* ∈ [0.5, 1]*.* Convergence of this propagation technique is guaranteed. We summarize the proof noted in [47] below for the sake of completeness.

The function above can be written at convergence as *S* = *VS + (1 – α) S*^*(0)*^, where *V = α W’*, which can also be rearranged into *S* = *(1 – α) (I - V)*^*−1*^ *S*^*(0)*^. For convergence to a unique, non-negative solution to be guaranteed, *(I − V)*^*−1*^ > 0 must hold.

#### Lemma 1.

Largest eigenvalue of *V* < 1. *W’* is a column-stochastic matrix. Per the Perron-Frobenius theorem, its eigenvalues ∈ [−1, +1]. Since *α* < 1, the largest eigenvalue (i.e., spectral radius) of *V* < *α <* 1.

#### Lemma 2.

*(I − V)*^*−1*^ exists and is non-negative. (*I – V*) is an M-matrix since its in the form *sI* – *B*, with *s* = 1 > 0, *s* ≥ largest eigenvalue of *B* (i.e., *V*) by Lemma 1, and *V* > 0. An M-matrix is inverse positive, hence (*I − V)*^*−1*^ > 0.

Convergence can also be achieved iteratively [43, 48], which we apply at a maximum of 350 iterations and is more commonly deployed with large PPI matrices for practical considerations. The value of *α* we pick is 0.8. Other values in the [0.6, 0.8] range have little effect on results.

### Upward mobility gene identification

The mobility status of a gene is determined by its rank before and after propagation. A gene’s rank is calculated according to its arithmetic average score across samples. For each gene *g*_*i*_ ∈ *G*,

$$ \mathrm{Initial}\kern0.17em \mathrm{score}\kern0.5em {IS}_i=\frac{1}{N}\sum \limits_{j=1}^N{S}_{ij}^{(0)} $$

and

$$ \mathrm{Final}\ \mathrm{score}\ {FS}_i=\frac{1}{N}\ \sum \limits_{j=1}^N{S}_{ij}^{\left(\infty \right)} $$

Let *RIS* and *RFS* be the lists of gene ranks in *IS* and *FS*, respectively, i.e., *RIS*_*i*_ = rank of *g*_*i*_ in sorted *IS* and *RFS*_*i*_ = rank in sorted *FS.* The mobility status of *g*_*i*_, *MS*_*i*_, is then calculated as the difference between *RIS*_*i*_ and *RFS*_*i*_ as:

$$ {MS}_i={RIS}_i-{RFS}_i $$

Since higher scores lead to a higher rank, and a higher rank has a lower value (i.e., rank 1, 2, … |*G*|), genes whose ranks improve because of propagation have positive *MS* values, and ones with lowered ranks (downward mobility) negative ones.

We then define upward mobility status according to two parameters: mobility *β* and rank threshold *T.*

$$ UMG=\left\{{g}_i\right|\ {MS}_i\ge \beta .\mid G\mid \wedge {RFS}_i\le T\kern0.5em \forall i\in 1,2,\dots \mid G\mid \Big\} $$

Mobility *β* value determines the minimum upward jump size a gene needs to make to be considered for UMG status. For instance, a *β* value of 0.1 in a PPI network with 10,000 nodes requires a gene’s position to improve by a minimum of 1000 ranks. We choose stringent values of *β* dictated by TCGA cohort size and the variance of each cancer type’s mutational. Cancer types with a high number of samples and/or a high variance of gene mutation frequency receive a value of 0.25 (BRCA, COAD, HNSC, LUAD, LUSC, PRAD, STAD, UCEC), others with moderate variance a value of 0.2 (CESC, KIRC, KIRP, LIHC) and 0.15 (ESCA, READ), and low variance and/or cohort size cancer types a value of 0.05 (CHOL, KICH, THCA). These values ensure that to be considered a UMG, a gene has to jump hundreds to thousands of ranks during propagation depending on the PPI network and cancer type under study. Rank threshold *T* specifies the minimum rank a gene needs to achieve after propagation to be considered a UMG. We choose *T* = 1000 to strictly focus on the top 10–16% of genes (i.e., approximately top 10% in STRING and top 16% in HumanNet v2), a threshold that has proved to be effective in other studies [20].

We further apply two optional selection criteria on the final UMG lists based on (i) each gene’s DepMap scores in CRISPR and RNAi experiments and (ii) propagation within multiple PPIs. Per (i), UMG becomes:

$$ UMG=\left\{{g}_i\right|\ {MS}_i\ge \beta .\left|G\right|\wedge {RFS}_i\le T\wedge {DM}_i\ge p,\kern0.5em i\in 1,2,\dots \mid G\mid \Big\}, $$

where *p* is the proportion of cancer type-specific cell lines in which a gene’s DepMap score is negative (i.e., its knockout has a negative impact on cancer cell survival), and *DM*_*i*_ is the maximum value across CRISPR and RNAi experiments. We choose *p* = 0.5 (50%), which ends up eliminating 2–10 out of 30–91 genes per cancer type (Additional file 3: Table S3). Per (ii), integration of lists across *K* PPI networks yields the intersection of lists. In this paper, to increase confidence in selected genes, we integrate lists over cancer type-specific STRING and HumanNet v2 networks. Formally,

$$ {UMG}_{\mathrm{Final}}={UMG}_{G_1}\cap {UMG}_{G_2}\cap \dots {UMG}_{G_K} $$

### Statistical validation of rankings

To assess the validity of ranking after propagation, we tested if known COSMIC genes are ranked significantly higher than other genes using the one-sided Mann-Whitney *U* statistical test (also known as one-sided Wilcoxon rank sum test). Results show a strong enrichment of COSMIC genes towards highly ranked genes for all PPI network-cancer type combinations (*p* < 10^−5^, Additional file 2: Table S2).

### Driver and COSMIC genes

Cancer type-specific driver genes were obtained from Bailey et al.’s except for COAD and READ which were combined into a single group in that study. For these two cancer types, we designated tissue-specific COSMIC v90 genes as the driver genes.

### UMG vs non-UMG comparisons

In the first set of comparisons, Mann-Whitney *U* one-sided test is used to compare the distribution of a percentage-based score of negatively impacted cell lines by UMGs vs non-UMGs in each cancer type. Each gene’s percentage-based score value is equal to the percentage of its negative DepMap scores among *k* cancer type-specific cell lines and the average of these values (to account for the distribution of DepMap scores across cell lines). To calculate a more stringent score and reduce false positives, we also assume the presence of at least one cancer cell line with a non-negative DepMap score, which especially accounts for cancer types with a small number of cell lines in the DepMap database. Hence, the score is the sum of each gene’s *k* + 1 values mentioned above divided by *k* + 2. An alternative hypothesis for each of the Mann-Whitney *U* tests is *H*_1_ = *ψ*(*UMG*) is shifted to the right of $\psi \left(\overline{UMG}\right)$, where *ψ*(*X*) is the percentage-based distribution of negatively impacted cell lines over genes in set *X*. Cancer type-specific cell lines are selected based on annotations provided in the DepMap dataset. For cancer types not represented among the cell lines in DepMap, we used values across all 750 (CRISPR knockout data) and 712 (RNAi) cell lines. A negative DepMap dependency score indicates decreased cell survival after gene knockout in a particular cell line. For RNAi experiments, we use data with enhanced batch and off-target processing as described in [41].

### UMGs vs gene candidates identified by other network methods

Hierarchical HotNet (HHotNet) generates statistically significant results (*p* < 0.05) in only 5 of the 17 cancer types after integrating its results for both PPI networks (HHotNet-consensus): ESCA, KIRC, LIHC, LUAD, and LUSC. As a result, we include HHotNet results from two other settings described below. In 13 cancer types, HHotNet generates statistically significant results for one of the two PPI networks, and in two others (PRAD and READ) significant result with a relaxed threshold (0.05 < *p* < 0.1). We include HHotNet results from both the largest subnetwork (HHotNet-LC) and all subnetworks with more than one node (HHotNet-all) in comparisons. Namely, for 15 cancer types, we choose results from STRING in BRCA, ESCA, HNSC, KICH, KIRC, LIHC, LUAD, LUSC, STAD, and THCA and from HumanNet v2 in CESC, COAD, PRAD, READ, and UCEC. In CHOL and KIRP, HHotNet results were not statistically significant for both PPI networks, so we exclude results for this method. In all runs, we execute HHotNet in default settings with 1000 permutations using the second controlled randomization approach suggested in [22]. For FDRNet, we run the method to detect subnetworks for all seed genes and in default settings. We convert MutSig2CV [45] *p*-values across TCGA cohorts to local FDR values using the scripts provided by FDRNet. We use FDRNet results for 16 cancer types over the STRING network as this method was not able to detect any subnetwork over HumanNet v2 for almost all seed genes (664/673, 98%). No FDRNet results could be produced for CHOL. In nCOP, we use lists of rarely mutated genes reported in [44] (Fig. 4) on the TCGA somatic mutational dataset in 15 of the 17 cancer types studied in our paper (all except CHOL and ESCA). For Zhou et al.’s propagation method, we select the top *k* genes identified post-propagation, where *k* is the equivalent number of UMGs for each cancer type across networks. In its edge-normalized setting, we divide each gene’s post-propagation score by the same score when propagation *α* = 1 (i.e., ignoring initial scores) before selecting top genes. For MutSig, we select all genes with statistically significant results (FDR < 0.1) across TCGA cohorts. As these methods do not primarily focus on long tail genes, we remove driver genes from these methods’ gene lists to ensure balanced comparisons with UMGs. It is worth noting however that including driver genes or the small percentage of UMGs filtered in the last step of the pipeline did not have a considerable impact on results (Additional file 5: Figs. S1-3).

### Enrichment analysis

Enrichment analysis to identify pathways, GO molecular functions, and GO biological processes is performed on g:Profiler [49]. Enrichment results with Benjamini *p*-adjusted < 0.05 are selected for analysis. Network visualization is executed using EnrichmentMap v3.0 on Cytoscape v3.8.2 [50], with a comprehensive subset of results related to cancer shown in Fig. 3. Frequent terms highlighted in red in Fig. 3b have ≥ 5 intra-cluster edges and those in Fig. 3c ≥ 10 edges. Frequent UMGs in Table 1 are identified based on their frequent presence in edges between a cluster’s nodes according (i.e., presence in ≥ 20 edges in Fig. 3b clusters and ≥ 30 in those of Fig. 3c).

### PPI analysis

Composite PPI is the union of high-quality edges in STRING v11 and HumanNet v2. The initial score of each gene is the one based on somatic mutations across a cohort as described earlier. Drivers are split according to initial score and degree with thresholds of 150 and 0.075, respectively. Initial scores of < 0.0015 are zeroed to attain lower FPR. Visualization and degree calculation are executed using Cytoscape v3.8.2.

### Manual literature curation of functionally validated UMGs

We manually cross-referenced each UMG with PubMed publications to detect which ones have been earlier reported to play a role in cancer based on functional experiments. We based results on an extensive search using the gene name AND “cancer” as keywords in PubMed. If any gene was the target of a previous functional assay, i.e., was deliberately overexpressed, suppressed, or mutated, and resulted an in vitro change in the proliferation or survival of cancer cell lines, it was annotated as functionally validated. Otherwise, the genes are considered not validated. Fully annotated UMG lists for each of the 17 cancer types are provided in Additional file 6: Table S5, while the list of publications associated with functionally validated UMGs is provided in Additional file 8: Table S7.

### Experiment validation: siRNA screening and annotation

Cell lines from the breast (MDAMB231, MDAMB468, BT549, HCC187), lung (H460, HCC1299), and prostate cancers (DU145) were cultured in RPMI medium supplemented with 10% HI-FBS and penicillin/streptomycin (1:100). The siRNA transfection experiments were performed at the Yale Center for Molecular Discovery. Reverse transfections were performed using 384-well tissue-culture treated plates (Corning CLS3764) pre-plated with siRNAs to achieve a 20-nM final assay concentration. RNAiMax transfection reagent (Invitrogen) was added to plates according to the manufacturer’s recommendations and incubated with siRNAs for 20 min. Cells were then seeded at plating densities optimized during assay development (MDAMB468, HCC1187, and BT549 seeded at 4000 cells per well; MDAMB231 and H460 seeded at 1000 cells per well; DU145 and HCC1299 seeded at 500 cells per well) and incubated at 37°C. After 72 h, CellTiter-Glo (Promega) was used to monitor viability. Each screening plate contained 16 replicates of negative siRNA controls (either siGENOME Smart Pool non-targeting control #1, #2, or #4, Dharmacon) and positive siRNA controls (siGENOME Smart Pool Human PLK1 or KIF11, Dharmacon). Signal-to-background (S/B), coefficient of variation (CV), and Z prime factor (Z’) were calculated for each screening plate using mean and standard deviation values of the positive and negative controls to monitor assay performance. All cell lines were obtained from ATCC and have been thoroughly tested and authenticated by the vendor. The cell lines will be routinely monitored for correct morphology and growth characteristics to confirm cell line identity. For each cell line, test siRNA data were normalized relative to the mean of negative control samples (set as 0% effect) and the mean of positive control samples (set as 100% effect). Three standard deviations of the negative control samples were used as a cutoff to define screen actives.

## Supplementary Information

**Additional file 1:** **Table S1.** Cohort Size and number of PPI network nodes after expression-based filtering

**Additional file 2:** **Table S2.** Statistical validation results of post-propagation rankings

**Additional file 3:** **Table S3.** Number of UMGs across cancer types and PPI networks

**Additional file 4: Table S4.** Statistical validation results of UMGs' negative impact on *in vitro* cancer cell line survival

**Additional file 5:** **Figs. S1-19.** Results before filtering steps and PPI network analyses of UMG-driver relationships across cancer types

**Additional file 6:** **Table S5.** Functional validation and text mining results associated with UMGs across cancer types

**Additional file 7: Table S6.** Results of *in vitro* experiments

**Additional file 8:** **Table S7.** References of functionally validated genes in **Table S5**

**Additional file 9:** Review history

## Data Availability

UMG detection code is available at https://github.com/gersteinlab/UMG [51] and 10.5281/zenodo.5500467 [52]. Results in the paper are in whole or part based upon data generated by the TCGA Research Network: https://www.cancer.gov/tcga. MC3 high-quality somatic mutation dataset is obtained from [53]. STRING v11 [54] and HumanNet v2 [55] functional network (FN) are respectively downloaded from https://string-db.org/ and https://www.inetbio.org/humannet. Gene expression data corrected for batch effect and study-specific bias are downloaded from RNAseqDB [56] at https://github.com/mskcc/RNAseqDB. Variant annotations are based on RefSeq hg19 provided via ANNOVAR 2018b [57], and gene length values are provided via the bioMart Bioconductor package [58]. Genetic dependency data from the Cancer Dependency Map [30] (for both CRISPR and RNAi experiments) are downloaded from https://depmap.org/portal/download/, MutSig2CV [45] data across cancer types from http://gdac.broadinstitute.org, COSMIC v90 census gene list from https://cancer.sanger.ac.uk/cosmic, and CancerMine v24 [59] gene lists from http://bionlp.bcgsc.ca/cancermine.
