# Supplementary material for: Network propagation-based prioritization of long tail genes in 17 cancer types
Source: Genome Biol. 2021 Oct 7;22:287. doi: 10.1186/s13059-021-02504-x (PMC8496153; doi:10.1186/s13059-021-02504-x)

Fig. S1 Impact on cancer cell line survival of UMG lists before the DepMap filtering step compared to other methods' lists. Extension to Figure 4.

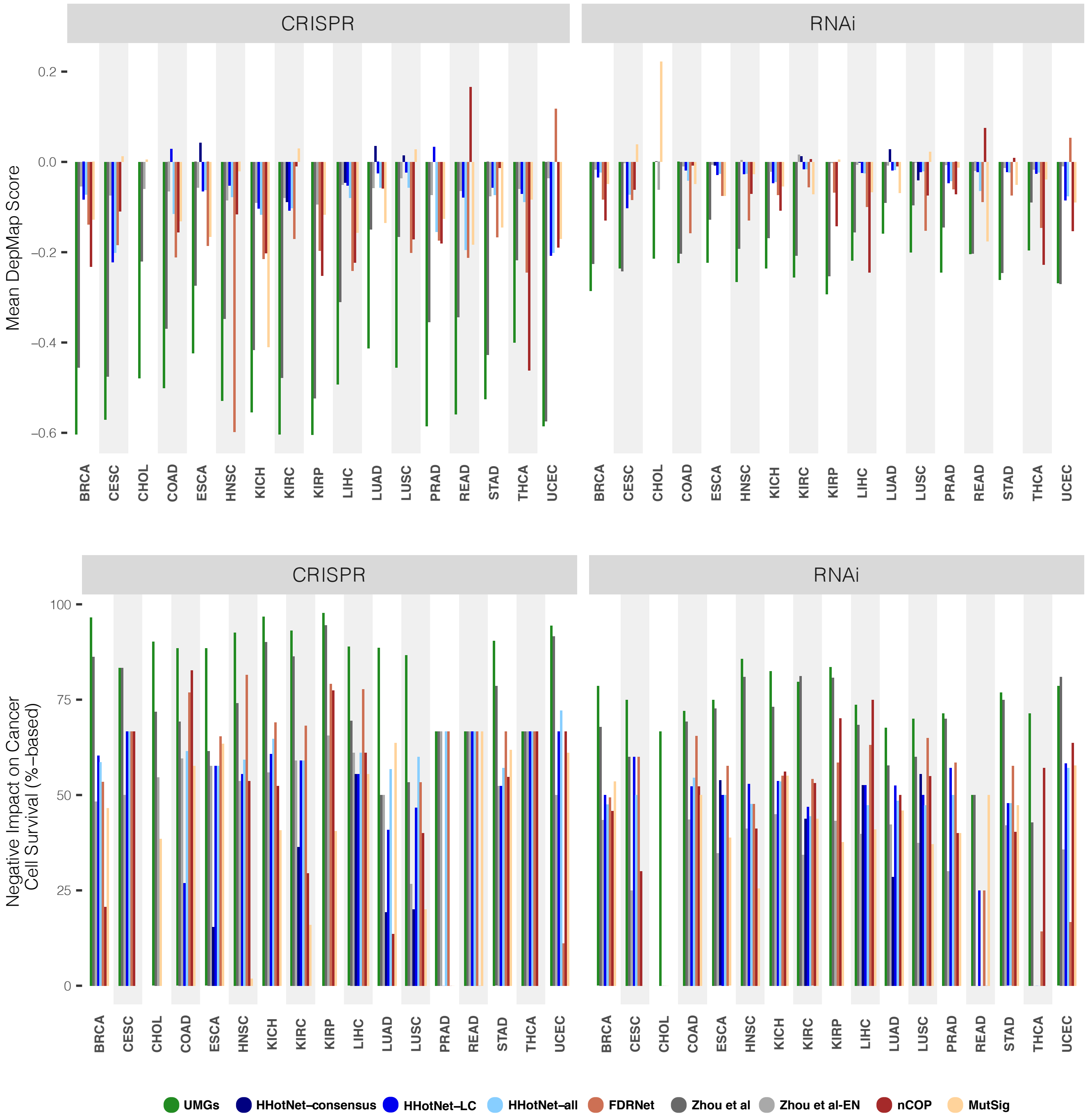

Fig. S2 Impact on cancer cell line survival of UMG lists compared to other methods' lists, all including known driver genes. Extension to Figure 4.

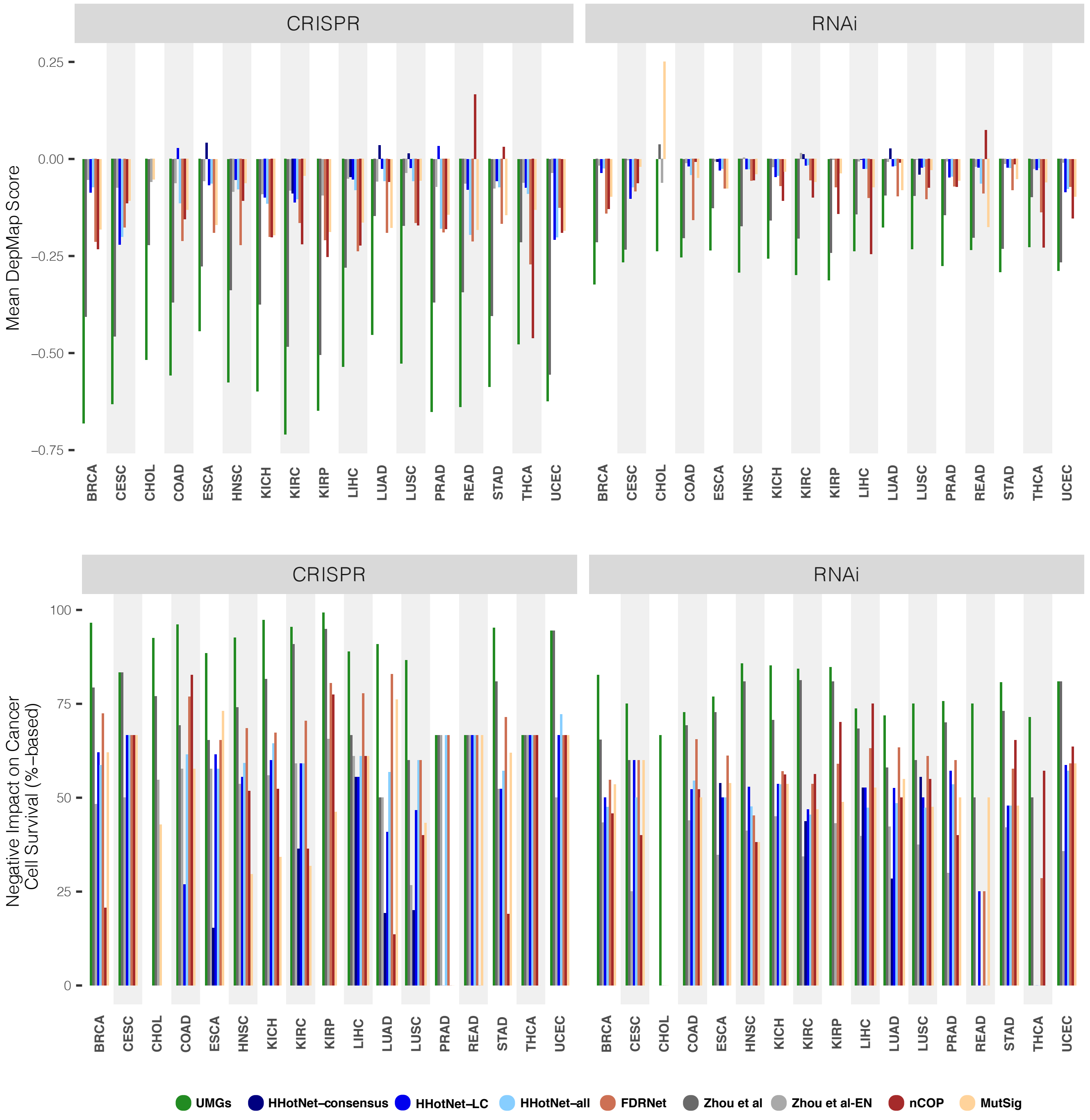

Fig. S3 Impact on cancer cell line survival of UMG lists before the DepMap filtering step compared to other methods' lists, all including known driver genes. Extension to Figure 4.

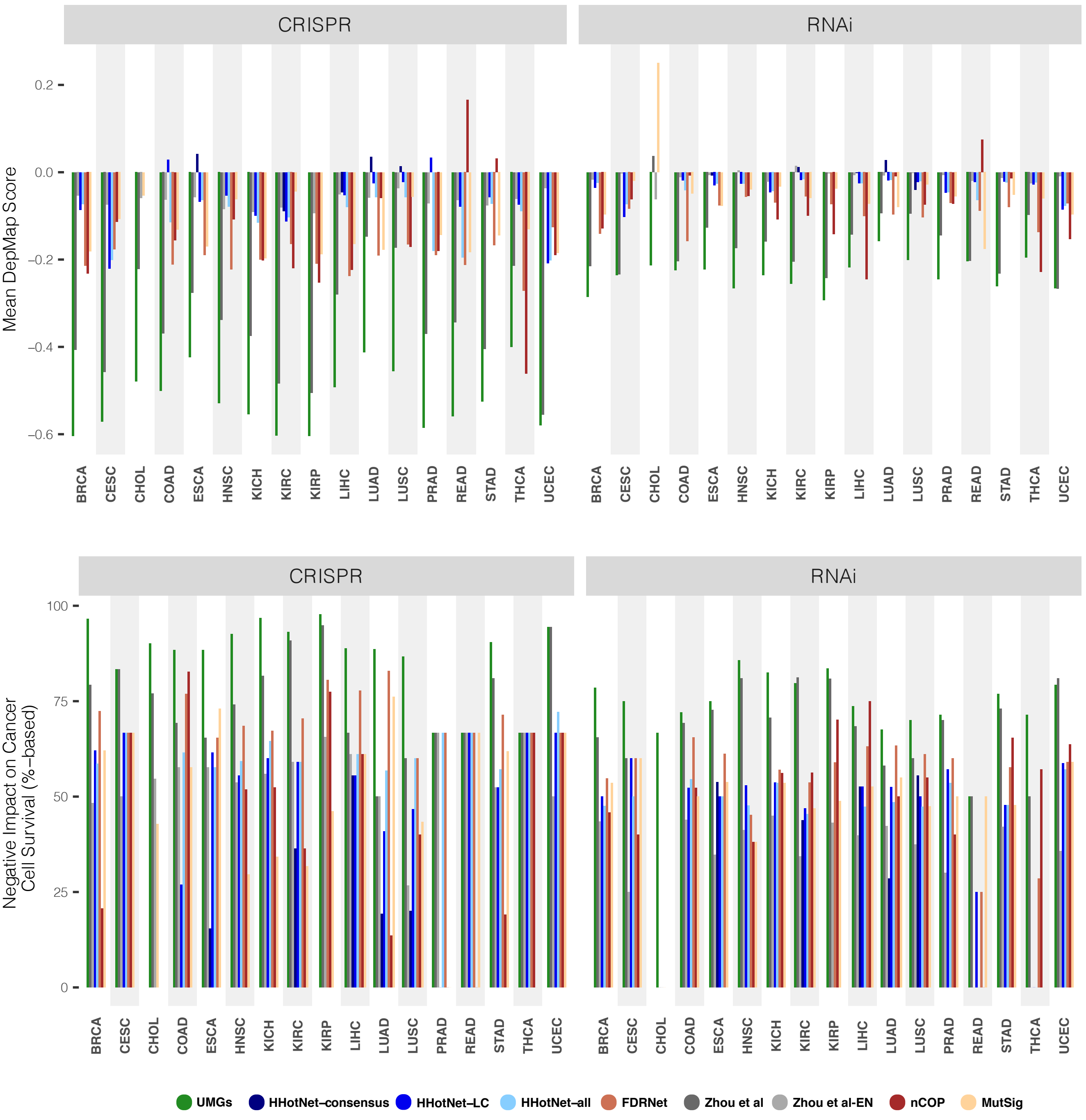

Fig. S4 PPI network analysis of the relationships between UMGs (white nodes) and known driver genes (red) in CESC. Extension to Figure 5.

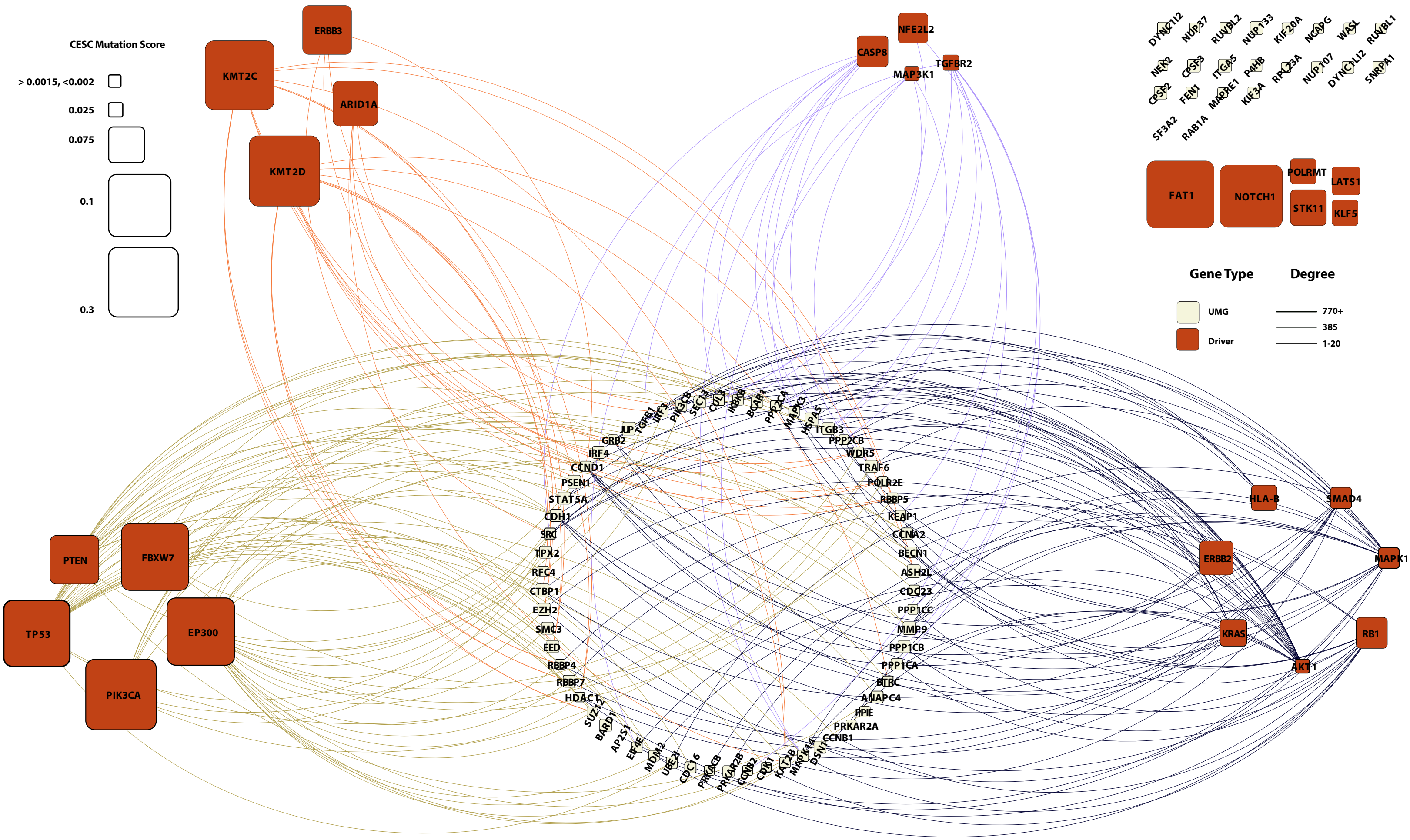

Fig. S5 PPI network analysis of the relationships between UMGs (white nodes) and known driver genes (red) in CHOL. Extension to Figure 5.

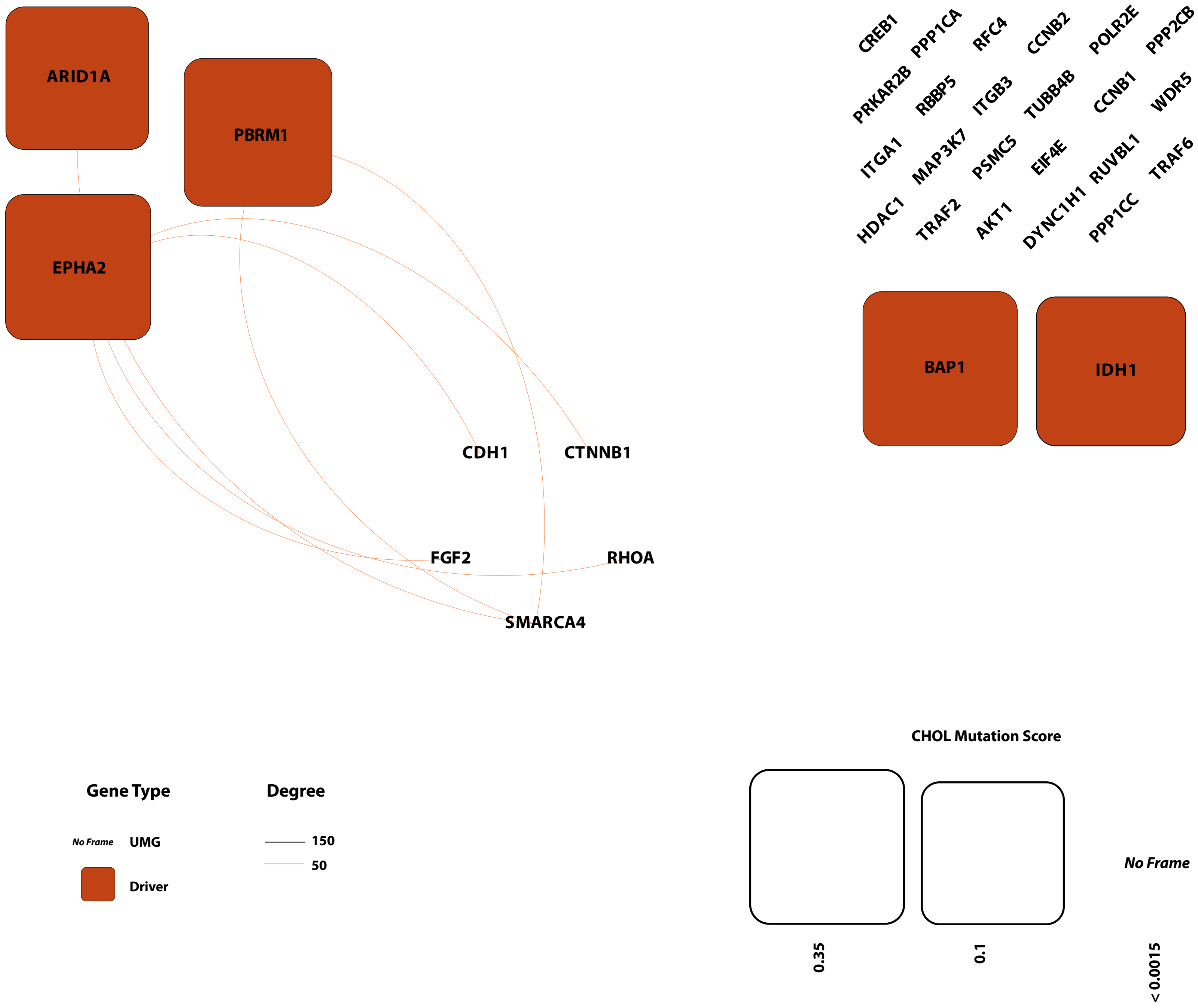

Fig. S6 PPI network analysis of the relationships between UMGs (white nodes) and known driver genes (red) in COAD. Extension to Figure 5.

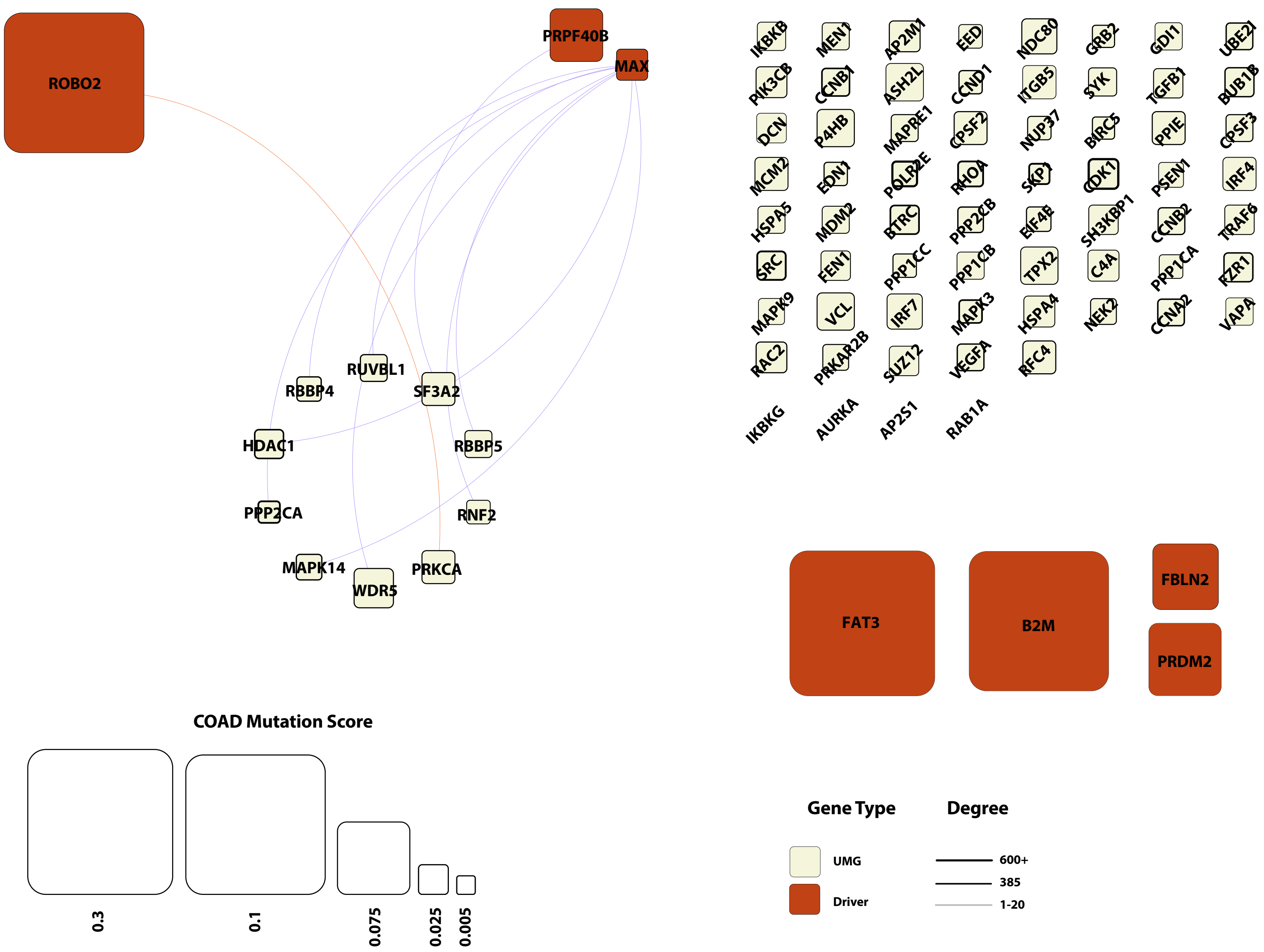

Fig. S7 PPI network analysis of the relationships between UMGs (white nodes) and known driver genes (red) in ESCA. Extension to Figure 5.

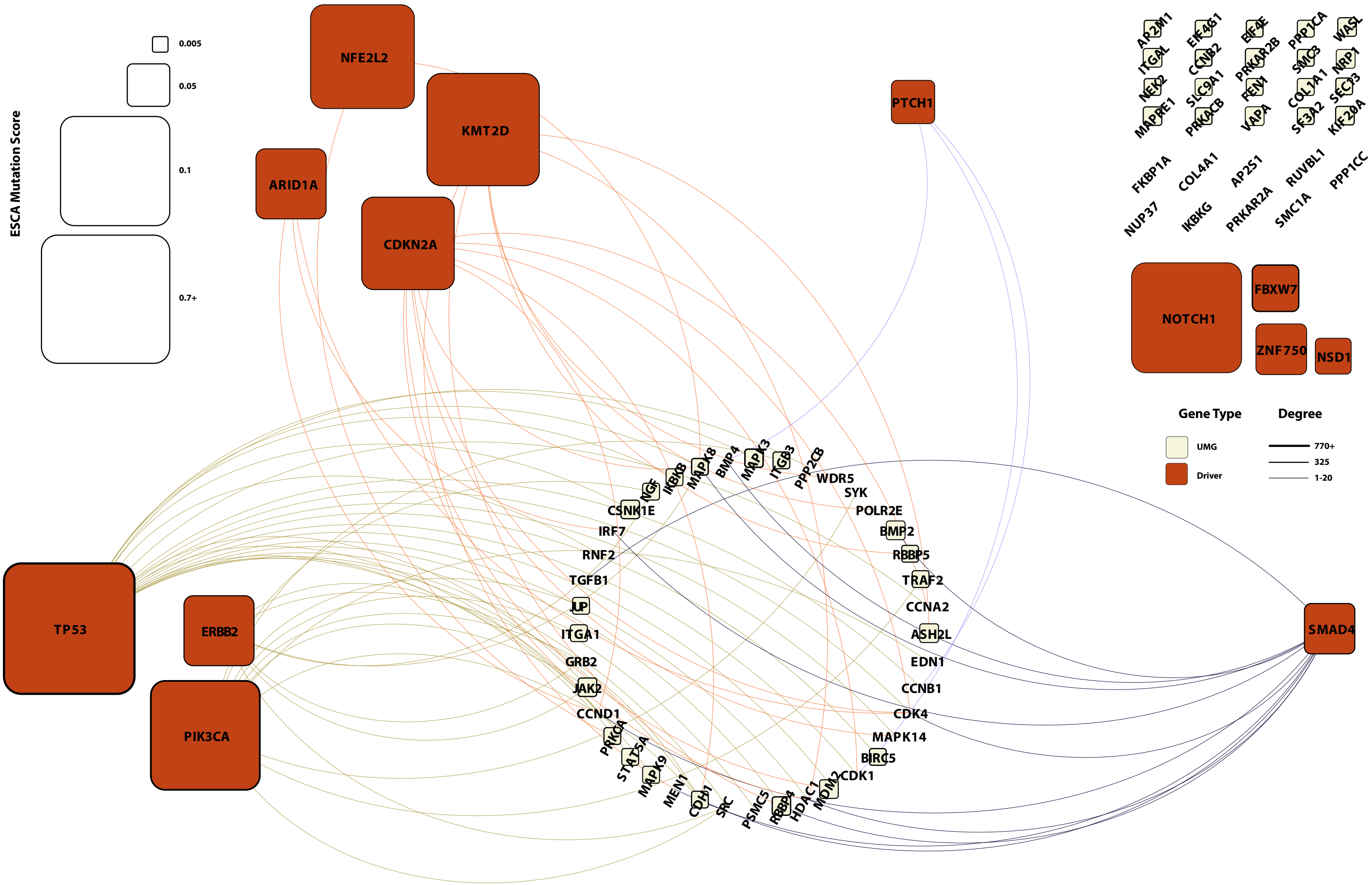

Fig. S8 PPI network analysis of the relationships between UMGs (white nodes) and known driver genes (red) in HNSC. Extension to Figure 5.

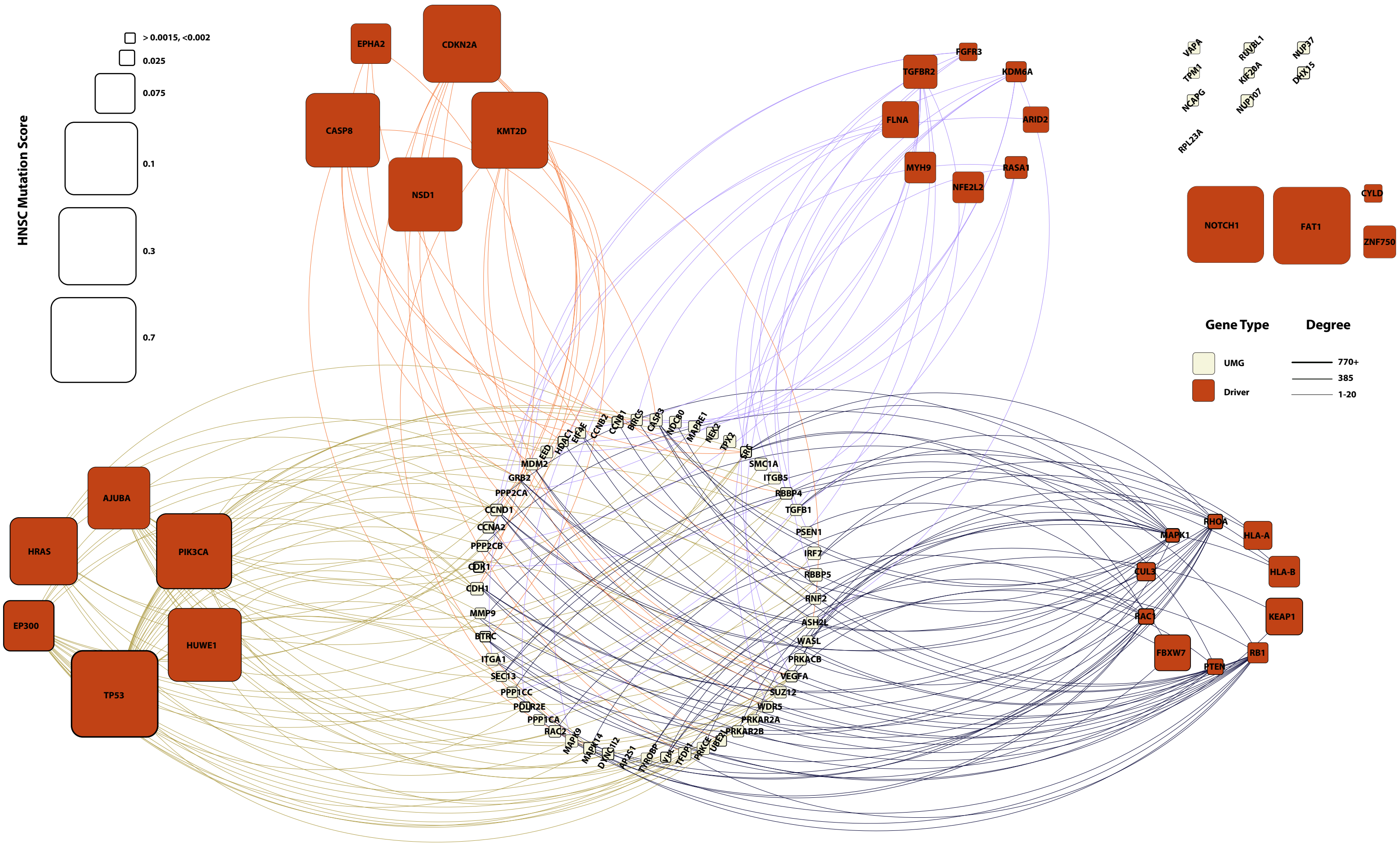

Fig. S9 PPI network analysis of the relationships between UMGs (white nodes) and known driver genes (red) in KICH. Extension to Figure 5.

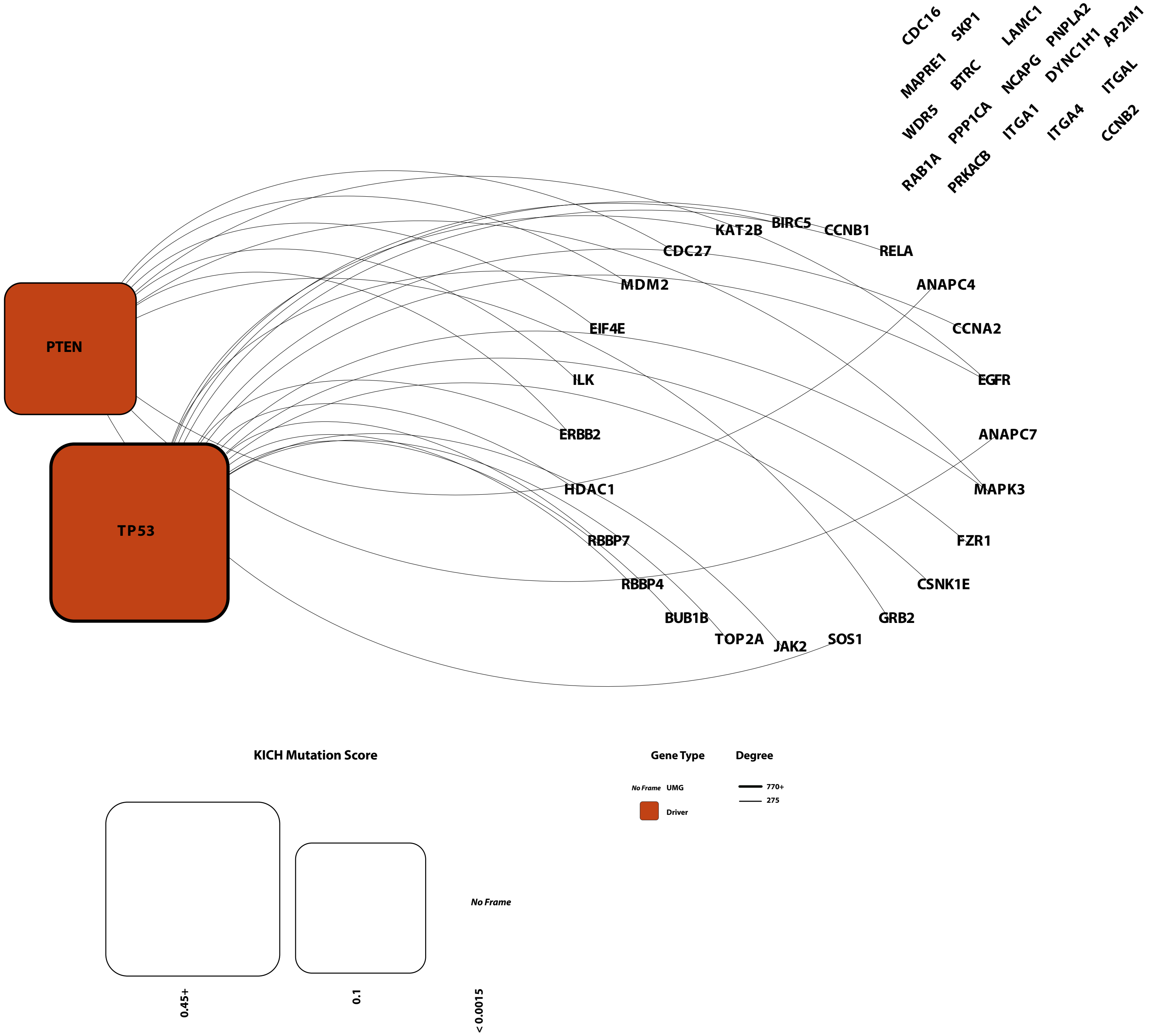

Fig. S10 PPI network analysis of the relationships between UMGs (white nodes) and known driver genes (red) in KIRC. Extension to Figure 5.

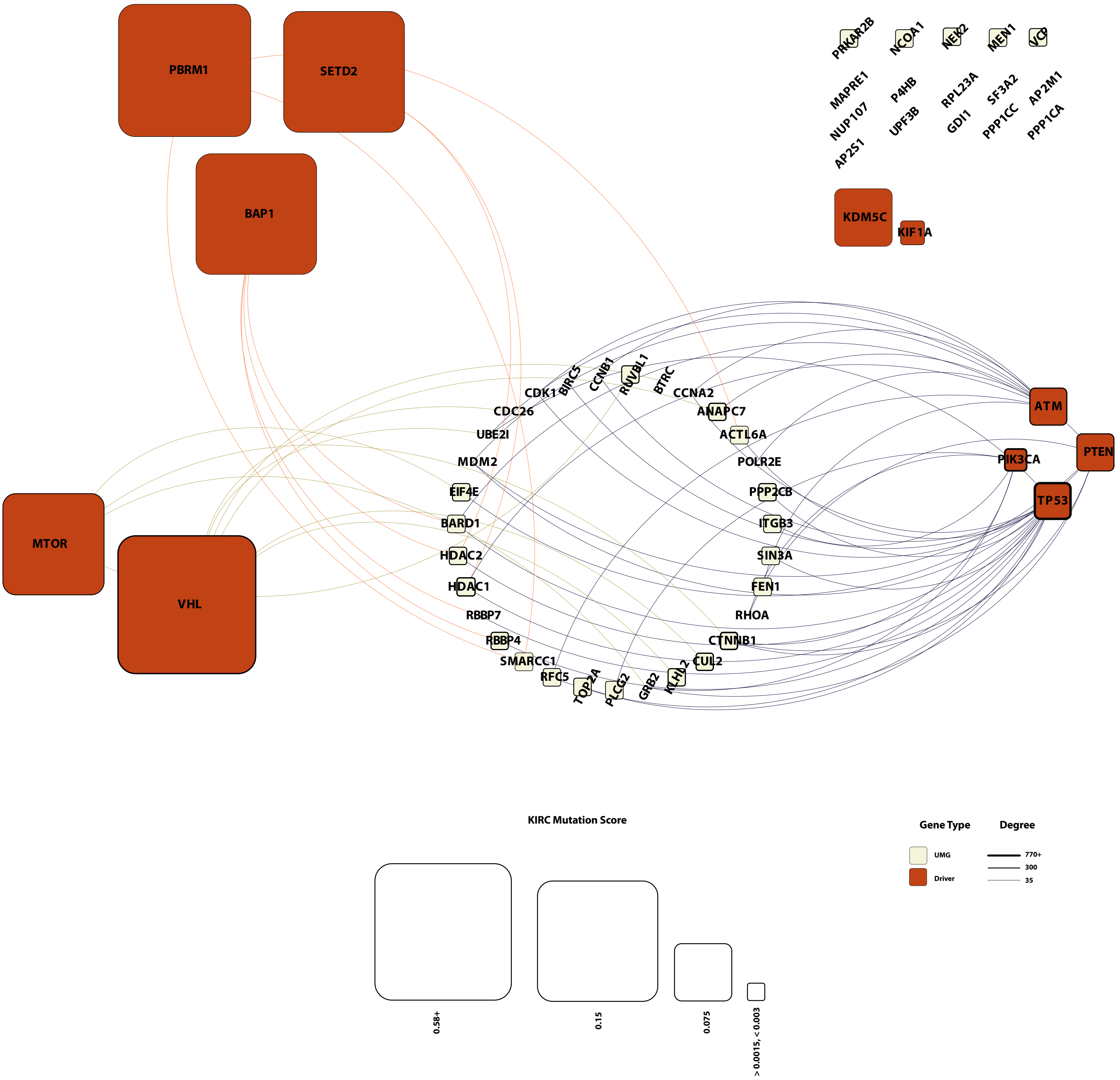

Fig. S11 PPI network analysis of the relationships between UMGs (white nodes) and known driver genes (red) in KIRP. Extension to Figure 5.

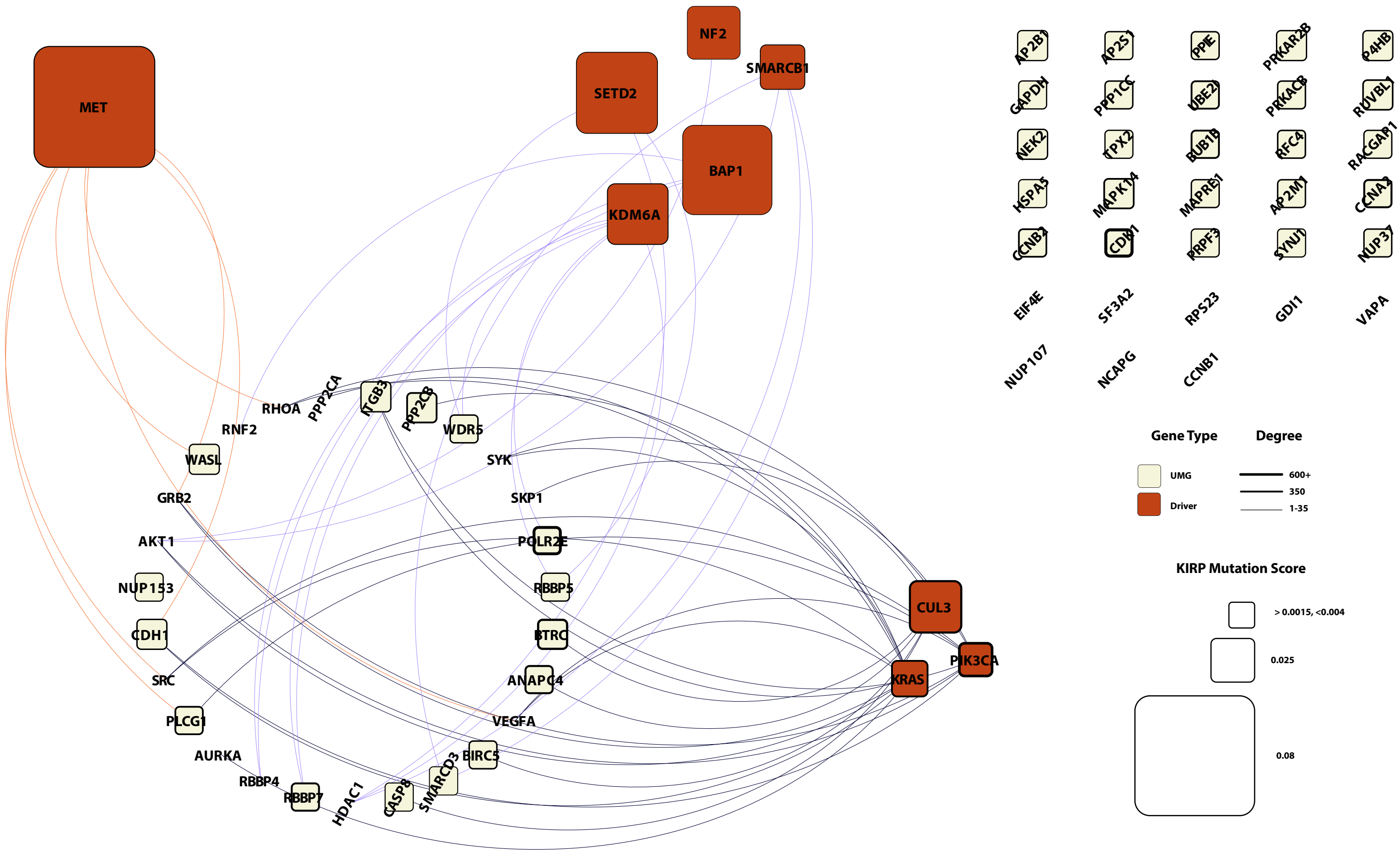

Fig. S12 PPI network analysis of the relationships between UMGs (white nodes) and known driver genes (red) in LIHC. Extension to Figure 5.

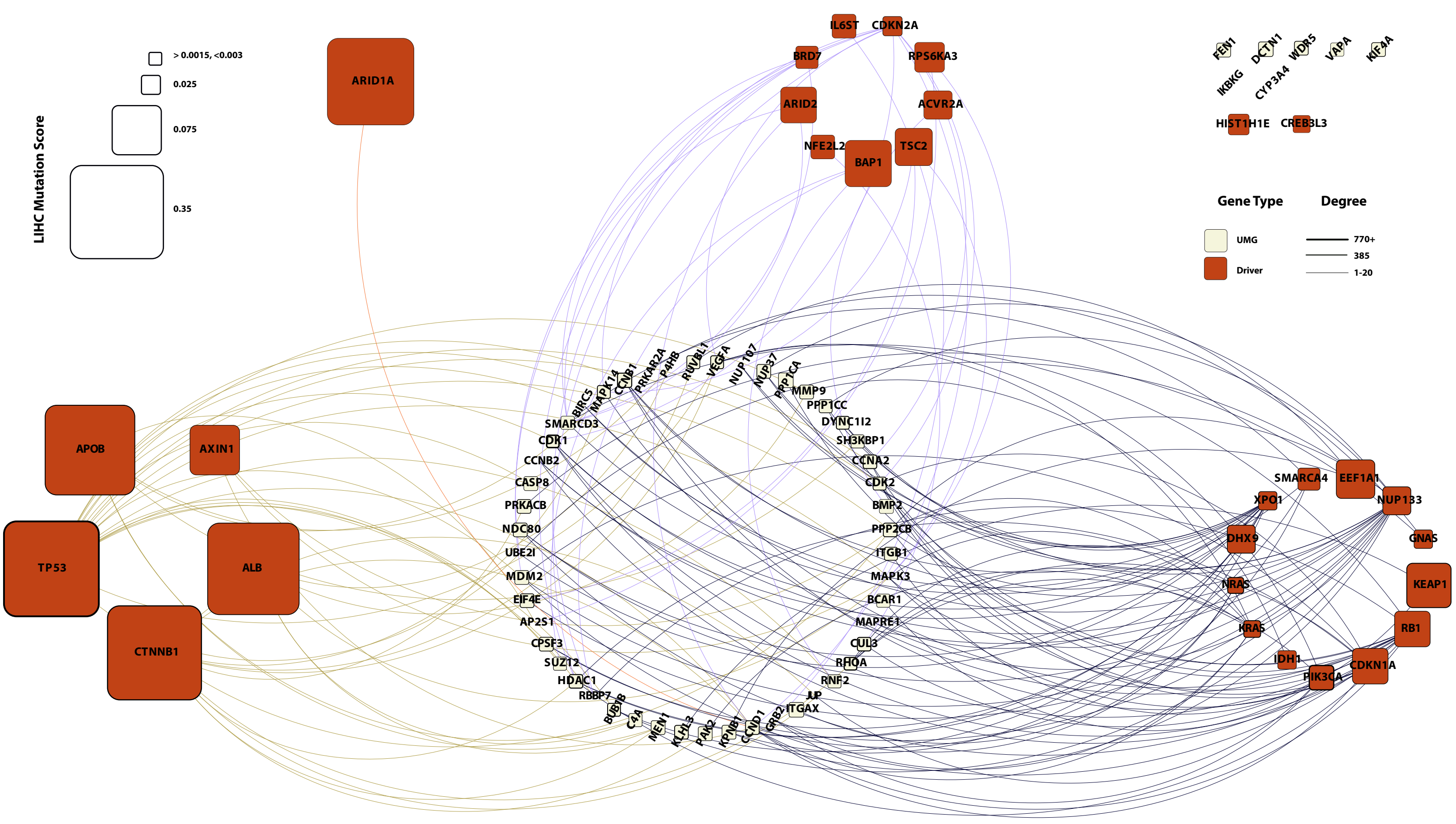

Fig. S13 PPI network analysis of the relationships between UMGs (white nodes) and known driver genes (red) in LUAD. Extension to Figure 5.

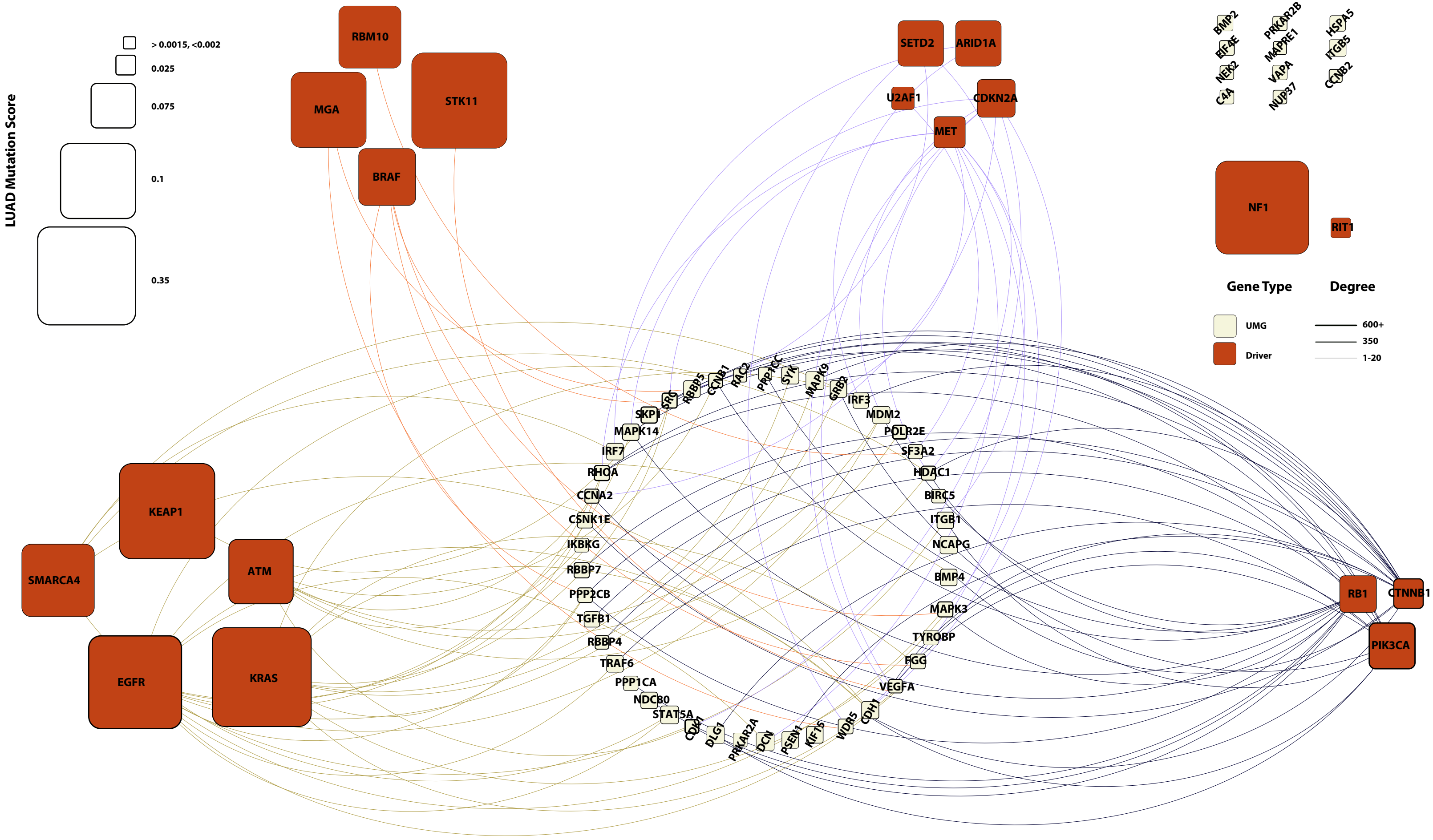

Fig. S14 PPI network analysis of the relationships between UMGs (white nodes) and known driver genes (red) in LUSC. Extension to Figure 5.

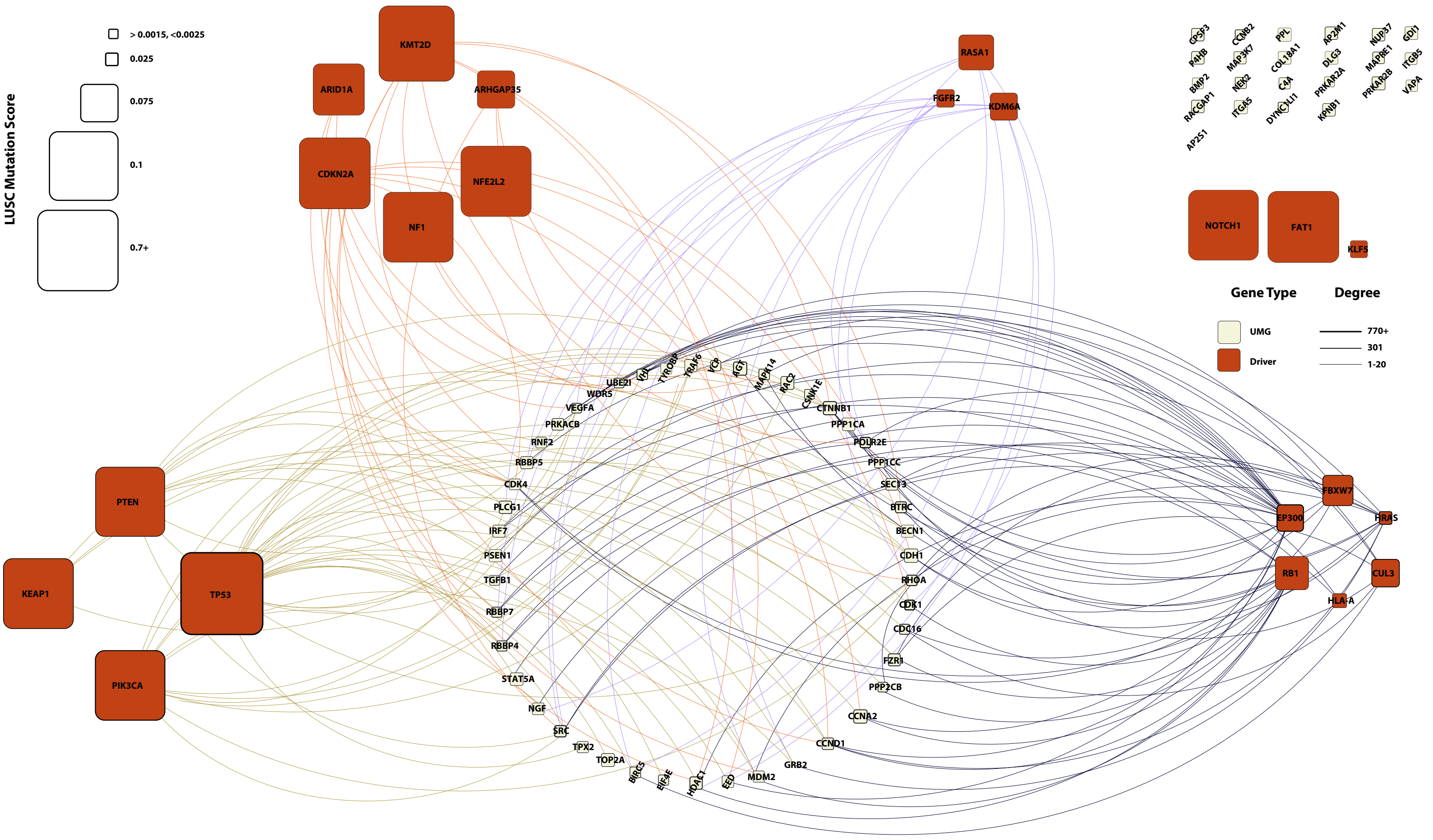

Fig. S15 PPI network analysis of the relationships between UMGs (white nodes) and known driver genes (red) in PRAD. Extension to Figure 5.

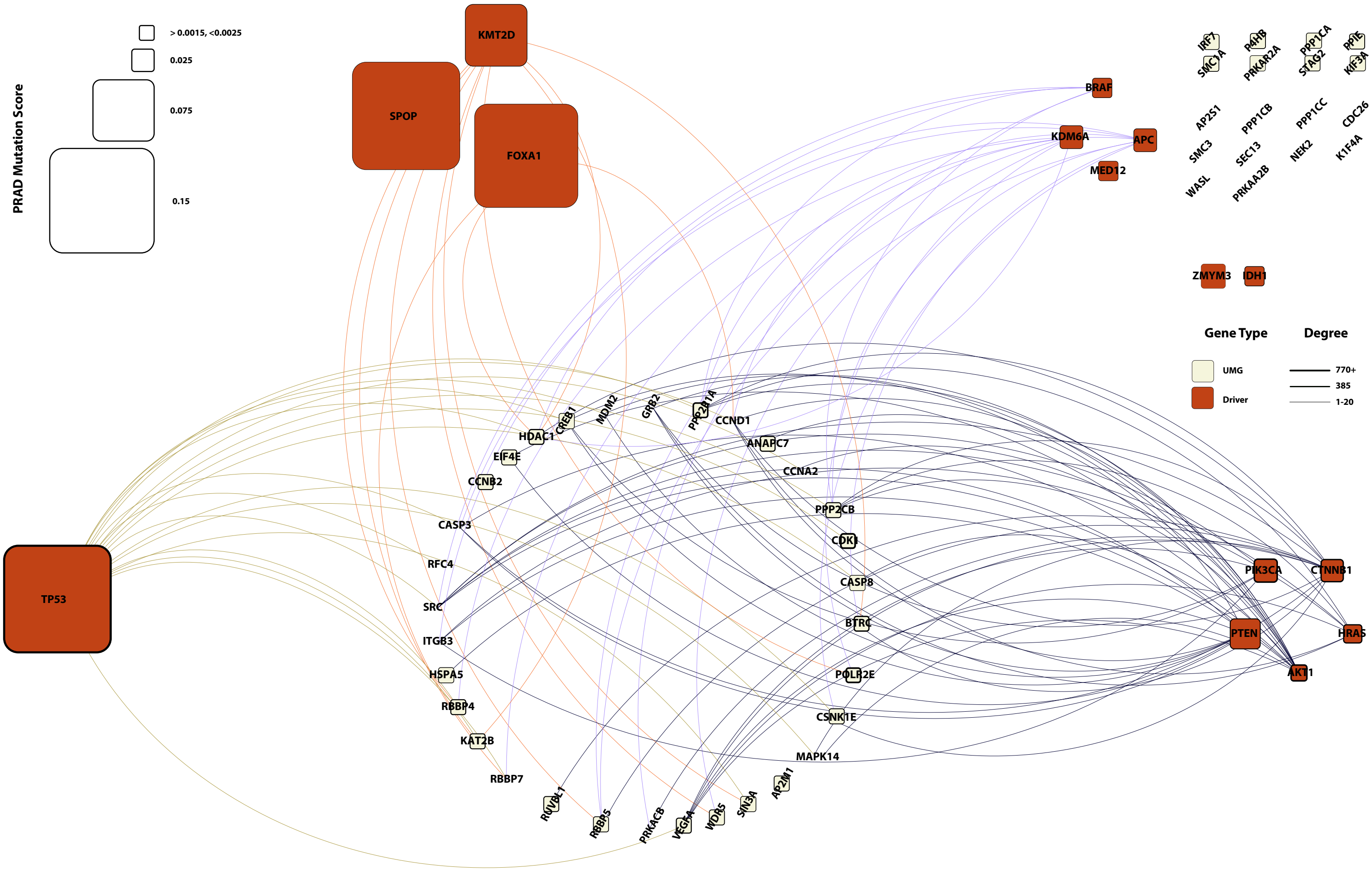

Fig. S16 PPI network analysis of the relationships between UMGs (white nodes) and known driver genes (red) in READ. Extension to Figure 5.

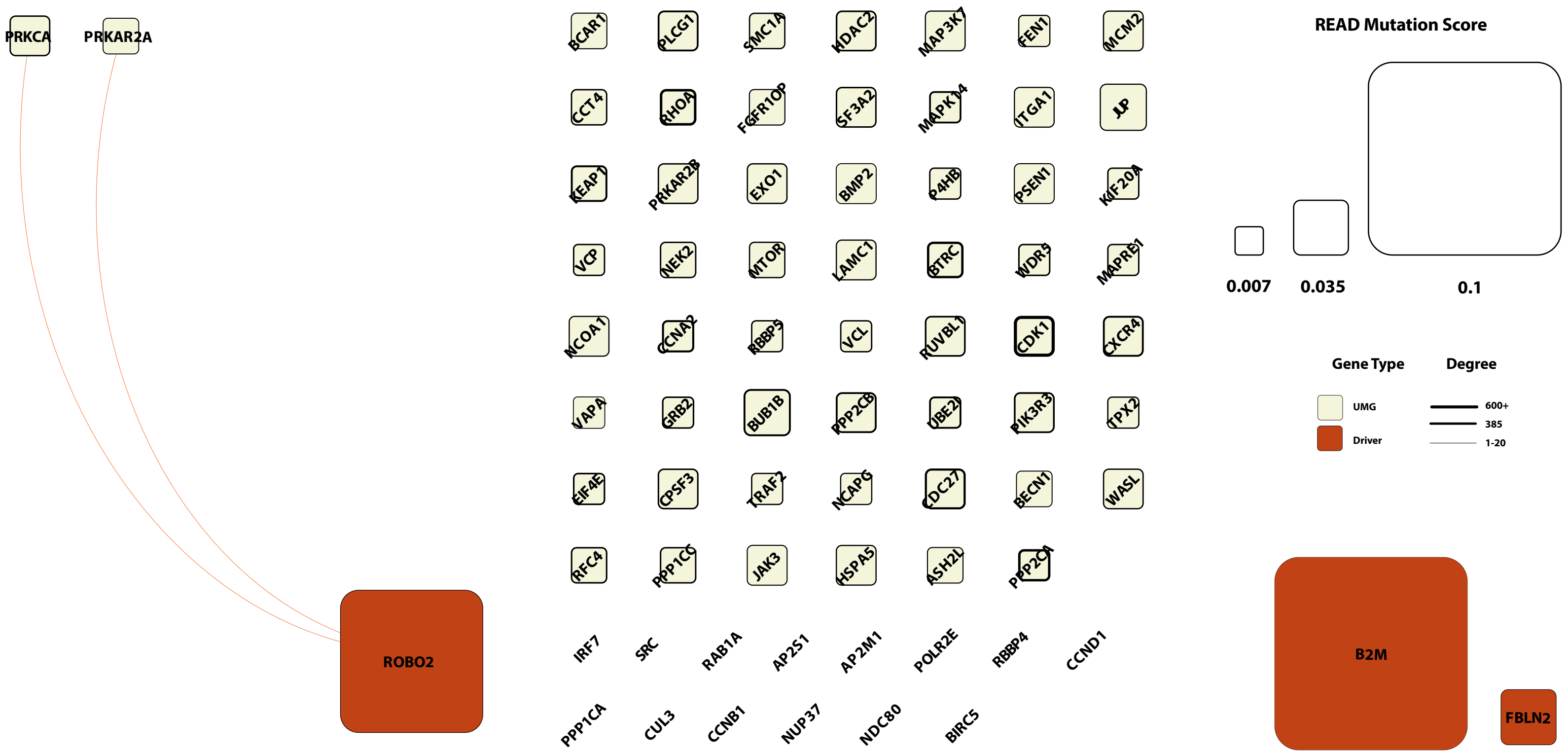

Fig. S17 PPI network analysis of the relationships between UMGs (white nodes) and known driver genes (red) in STAD. Extension to Figure 5.

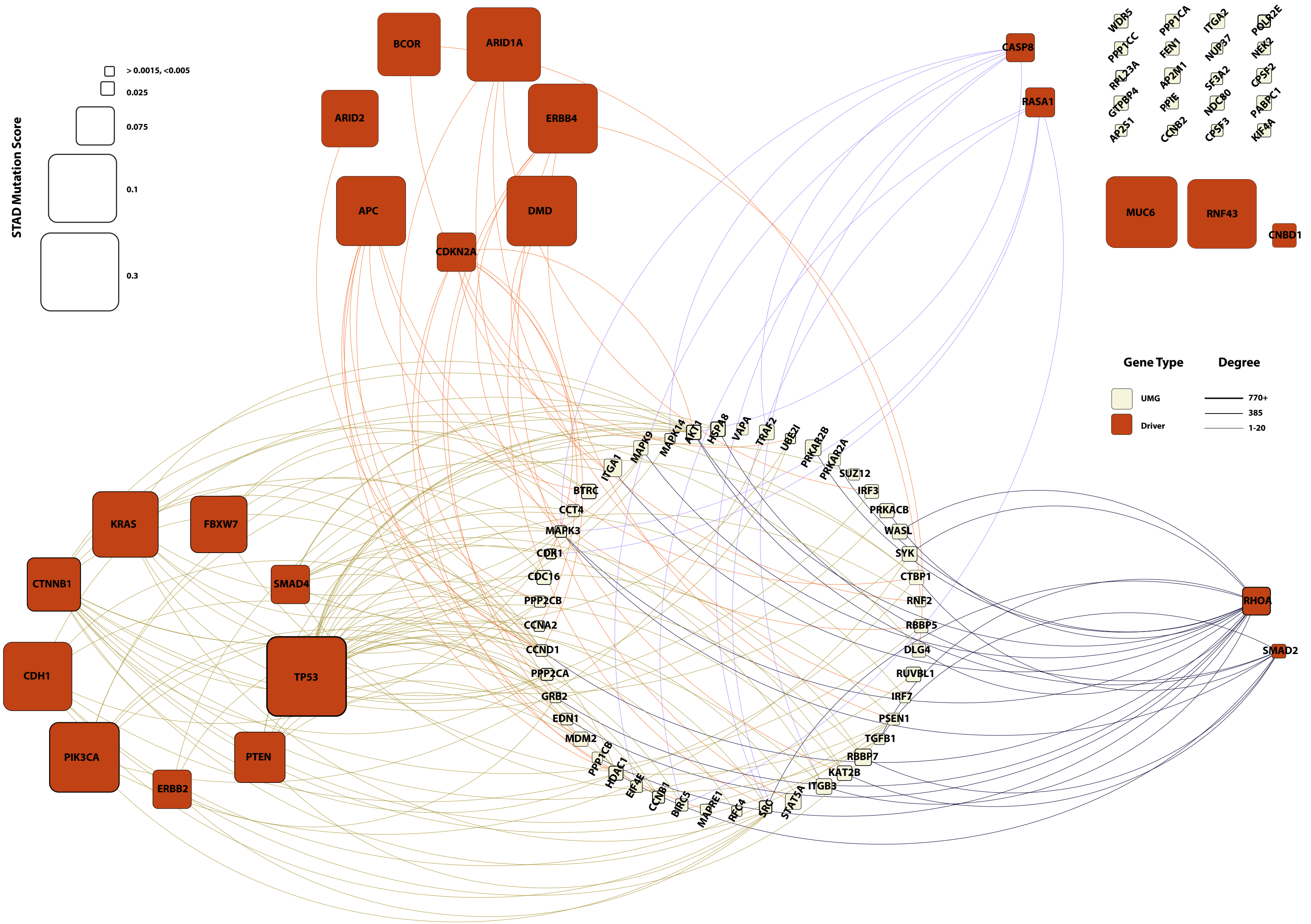

Fig. S18 PPI network analysis of the relationships between UMGs (white nodes) and known driver genes (red) in THCA. Extension to Figure 5.

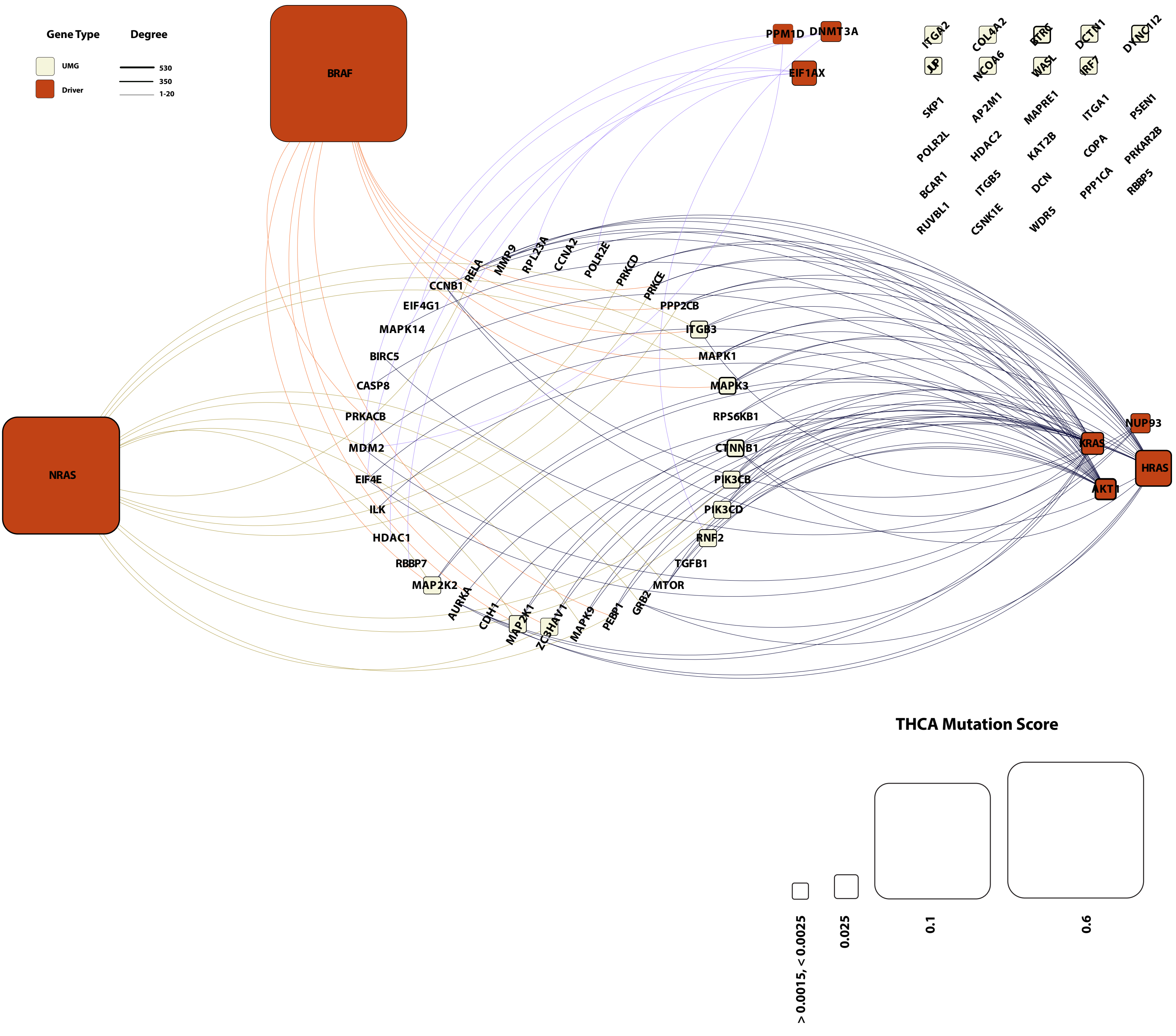

Fig. S19 PPI network analysis of the relationships between UMGs (white nodes) and known driver genes (red) in UCEC. Extension to Figure 5.

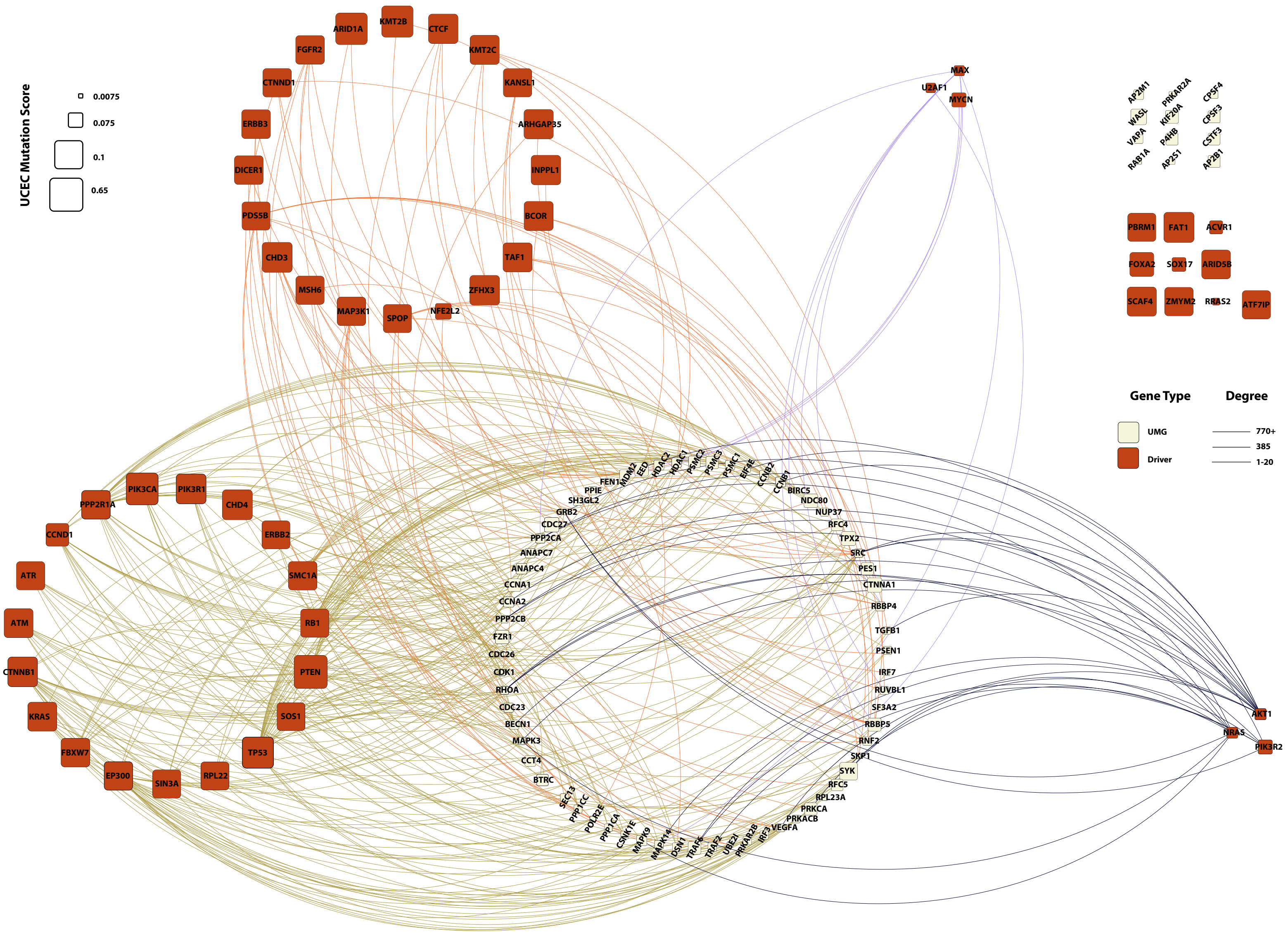

Supplement: Supplementary file 5 — Additional file 5: Figs. S1-19. Results before filtering steps and PPI network analyses of UMG-driver relationships across cancer types [file 13059_2021_2504_MOESM5_ESM.pdf]
