# Supplementary material for: Network propagation-based prioritization of long tail genes in 17 cancer types
Source: Genome Biol. 2021 Oct 7;22:287. doi: 10.1186/s13059-021-02504-x (PMC8496153; doi:10.1186/s13059-021-02504-x)
Supplement: Supplementary file 9 — Additional file 9: Review history [file 13059_2021_2504_MOESM9_ESM.docx]

Review History

**First round of review**

**Reviewer 1**

**Are you able to assess all statistics in the manuscript, including the appropriateness of statistical tests used?** Yes, and I have assessed the statistics in my report.

**Comments to author:**

This paper suggests an appealing concept of UMG genes whose mutation score increases after propagation. It is thus potentially different from classical propagation works

in that a gene will be highlighted only if it increased its score from before to after propagation, and not if it "simply" has high score.

However, this important distinction from earlier work is not sufficiently demonstrated in the paper. To do so, the authors need to:

1. exclude from analysis genes whose mutation score is very low to begin with, as by definition such genes will be part of the UMG set if their final score is high.

2. compare to one of the previous propagation methods that work on single genes (as opposed to the works compared to here that focus on identifying modules and not single genes).

Other comments:

- The use of humannet does not seem to contribute much - this is a very small network that focuses on functional links and covers only half of human genes.

- Even for string - the title suggests that the network will be a PPI network but in fact a functional network is used. It could be interesting to separate string into PPI and other functional relations and use these two networks instead.

- There is no justification for the thresholds used by the authors to filter edges in string or humannet or TCGA expression data.

- In 4.7 - a better statistical test here would be Wilcoxon rank sum test which will avoid the need of selecting a rank threshold.

**Reviewer 2**

**Are you able to assess all statistics in the manuscript, including the appropriateness of statistical tests used?** Yes, and I have assessed the statistics in my report.

**Comments to author:**

The study by Hussein Mohsein and colleagues uses network analysis in the TCGA dataset to nominate new cancer genes called UMGs (for Upward Mobility Genes). They use the technique of network propagation on gene interaction networks and prioritize genes whose mutation-based ranking becomes stronger once their network context is taken into account through this propagation. They validate their predictions using existing shRNA and CRISPR screens and also present new in vitro validation data but the latter part is underdeveloped. Overall the study is well executed and written and the network-driven approach is interesting as a build-on to existing network propagation method. Some comments below.

1. Is the network topology of UMGs contributing to their nomination in the cancer-type specific interaction networks? For example, are they hubs in the network or do they have few interaction partners? Are they on frequently used paths in the network?

2. How would the method perform on randomly permuted data, for example if mutation counts of genes were to be shuffled around in the networks ? This would help evaluate the false positive rates of such network-informed cancer gene discovery.

3. The authors use computationally predicted interaction networks STRING and HumanNet2. While these networks provide a larger search space, their computational origin makes them less reliable and the edges in the network may be difficult to interpret. How robust are their findings to those effects - can they recover the cancer genes if they focused on experimentally defined protein-protein interactions instead?

4. The study focuses on protein-coding mutations, but is it extendable to non-coding mutations? A recent study of the PCAWG used network and pathway-based approaches to nominate cancer genes in the long tail, including network propagation techniques.

5. The experimental validation data is described only in 10 lines and therefore not as convincing. How were the genes selected - it seems that some were the genes they predicted and others were somehow related genes? What controls were used? Is it expected to see such level of UMGs and others being validation? This part would substantially increase the value of the entire study in the opinion of the reviewer, but its current level of development is too limited to add this value.

6. The gene set enrichment analysis is performed using the DAVID software that employs databases that are more than five years old. The authors are likely to miss out on a substantial fraction of functional annotations of their UMGs.

**Authors Response**

**Point-by-point responses to the reviewers’ comments:**

### Reviewer #1

| Reviewer  Comment | This paper suggests an appealing concept of UMG genes whose mutation score increases after propagation. It is thus potentially different from classical propagation works  in that a gene will be highlighted only if it increased its score from before to after propagation, and not if it "simply" has high score. However, this important distinction from earlier work is not sufficiently demonstrated in the paper. To do so, the authors need to:  1. Exclude from analysis genes whose mutation score is very low to begin with, as by definition such genes will be part of the UMG set if their final score is high. |
| --- | --- |
| Author  Response | We thank the reviewer for these comments. We have updated the manuscript to highlight the point s/he raises on rankings, which helps distinguish our approach from classical propagation ones.  In the network analysis we performed in section 2.6, we created a specific category for UMGs with very low initial score that the reviewer refers to. These genes tend to be a small subset of UMGs in most cancer types. Given their initial low score, we classified them as either potential drug targets (rather than “weak drivers”) or potential false positives. We have updated the manuscript to clarify these points and to point them out in the corresponding figures. |
| Excerpt(s) from Updated Manuscript | **[1, paragraph 7] *“These genes are not merely pinpointed based on their high post-propagation ranks, but rather on the strong improvement in their pre- and post-propagation ranking difference that exceeds stringent measures. Hence, we describe these genes throughout this paper as upward mobility genes (UMGs). To the limits of our knowledge, this is the first propagation approach that focuses entirely on long tail genes.”***  **[2.6, paragraph 3] “*The third category includes UMGs with nearly no observed mutations in the TCGA cohort (i.e. very low initial score). These UMGs often form a small subset and are likely to be drug targets or false positives limited by the size of the cohort under study. In Figure 5 (and Supplementary Figures 3-18), they can be distinguished by their lack of node border (e.g. 6 genes in Figure 5: NUP37, UBE21, POLR2E, IRF7, BIRC5, and EIF4E).”*** |

| Reviewer  Comment | 2. Compare to one of the previous propagation methods that work on single genes (as opposed to the works compared to here that focus on identifying modules and not single genes. |
| --- | --- |
| Author  Response | We added comparisons with Personalized Page Rank (PPR), one of the most popular propagation methods underlying several single-gene studies (e.g. PMID 21536720). We also added comparisons with MutSig as a single-gene non-propagation method. Results continue to demonstrate a considerably higher impact of UMGs on cell line survival across cancer types. Manuscript, Figure 4, and Supplementary Figures (1 and 2) have been updated to reflect these additions. |
| Excerpt(s) from Updated Manuscript | **[2.4, paragraph 3] *“To have a better understanding of the specifications of UMGs, we compared their impact on the survival of cancer cell lines to that of genes selected by five other methods. Three of these methods are propagation-based and include FDRNet [43], Hierarchical HotNet (HHotNet) [22]—in 3 different settings, and personalized PageRank (PPR) [44]. The other two include nCOP [45], a non-propagation network method that recently demonstrated an ability to uncover non-driver genes across multiple cancer types, and MutSig [46], which identifies genes mutated more often than expected in a given cohort.”***  **[4.10, paragraph 1] “*For personalized PageRank (PPR), we select the top k genes identified post-propagation, where k is the equivalent number of UMGs for each cancer type. For MutSig, we select all genes with statistically significant results (FDR < 0.1) across TCGA cohorts—obtained for MutSig2CV from http://gdac.broadinstitute.org/.”*** |

| Reviewer  Comment | The use of humannet does not seem to contribute much - this is a very small network that focuses on functional links and covers only half of human genes.  Even for string - the title suggests that the network will be a PPI network but in fact a functional network is used. It could be interesting to separate string into PPI and other functional relations and use these two networks instead. |
| --- | --- |
| Author  Response | We thank the reviewer for this comment. We detail below the experiments we conducted to address the reviewer’s comment on information channels and our rationale behind selecting STRING and HumanNetv2. HumanNetv2 and STRING are two of the networks that rank high in a recent comprehensive assessment of more than 20 molecular networks in disease discovery by Huang *et al.* (PMID 29605183). In addition, they are two of the popular networks deployed in network-based studies in studying functional cancer genomics, and most recently COVID-19 (e.g. PMIDs 24037242, 33434495, 34016094*,* 33947863, and 33684134). The availability of this high number of networks poses a challenge on network-based research. A number of studies as a result opt to use a single network in their analyses. We chose for our analysis to be part of the more comprehensive studies that rely on multiple networks with a goal to reduce false positive rate in prioritizing long tail genes—an emerging research focus itself. The HumanNetv2 we use (HumanNetv2-FN) originally covers 17.2K human genes ([http://www.inetbio.org/humannet/about.php).](http://www.inetbio.org/humannet/about.php) In our analyses, we implemented a number of stringent node and edge filtering steps for the same FP rate reduction goal. After retaining high quality edges, gene expression-based filtering, and selection of nodes in the largest connect component, this number becomes 6.4-9.4K genes in cancer type-specific networks. The same applies for STRING, with a final range of 11.6-12.6K genes down from 19.3K in the original STRINGv11 network before filtering. The number of reported physical interactions has certainly increased to considerable levels during the last 10 years. In parallel, functional links do represent a valuable source to networks, and in many cases the majority of edges (as with HumanNetv2). We adopted in the manuscript a common rhetoric in the literature that broadly defines protein-protein interactions to encapsulate both physical interactions and functional associations, as these information channels are often used together (e.g. PMIDs 30476243, 24037242, and 34016094). We have updated the manuscript to clarify this point raised by the reviewer, and would be happy to explicitly describe the networks as functional. As the reviewer requested, we have also assessed UMG detection after removing functional edges from STRING using BRCA, ESCA, and LUAD as examples with well-size cohorts. Results demonstrate a strong association between the number of high quality edges, with or without functional ones, and the number of recovered genes from the UMG lists we report in the manuscript. For STRINGv11, we removed all functional information channels corresponding to co-expression, fusion, neighborhood, co-occurrence, and text mining. We retained the experiments and database channels. The latter admittedly presents a hybrid score of curated physical interactions via protein complex membership and other predicted associations. Yet, as these are comprehensive interactions that cannot be separated from predicted ones in the single score provided for this channel in STRING, and given the considerably small size of edges in the experiments channel alone (7%), we decided to retain both channels and exclude all others that make the bulk of functional edges. We then recalculated edge confidence scores according to the Bayesian correction approach used in STRING (PMID 15608232). The total number of high quality undirected edges decreased after removing functional channels by **19.1%** (from 419.7K to 339.4K), and that of nodes by 14.7% (from 17.1K to 14.6K). Given this reduction in the number of edges, we hypothesized that for a consistent UMG detection, the recovery of UMGs we reported in the manuscript should follow a comparable—and not bigger—reduction to that of high quality edges. Results confirm this hypothesis where respectively for BRCA, ESCA, and LUAD, 84.4, 80.2, and 83.7% of UMGs have been recovered for the reduced STRING network. For completeness, we list the number of UMGs we obtained on complete and reduced STRING networks for BRCA, ESCA and LUAD below. **Cancer Type** \| **UMGs in Complete Network List** \| **Recovered UMGs in Reduced Network List** \| **Recovery Rate (%)** \| **Reduction Rate (%)**; BRCA \| 527 \| 445 \| 84.4 \| 15.6; ESCA \| 566 \| 454 \| 80.2 \| 19.8; LUAD \| 498 \| 417 \| 83.7 \| 16.3 In sum, the UMGs we report in our manuscript can be predominantly captured by high quality edges across different channels, and the addition of functional links seems to boost the effectiveness of UMG detection. |
| Excerpt(s) from Updated Manuscript | **[Abstract, paragraph 1] *“The study of cancer-relevant genes often requires integrative approaches pooling together multiple types of biological data. Network propagation methods have demonstrated high efficacy in achieving this integration.”***  **[4.3, paragraph 1] *“We adopt the broad definition of protein-protein interactions that encompasses direct physical interactions alongside indirect functional ones derived from co-expression, gene fusion, text mining, co-essentiality, and pathway membership datasets among others.”***  **[3, paragraph 3] *“The incorporation of additional functional genomics data (e.g. noncoding mutations and methylation data), coupled with improvements in the accuracy of reported PPIs, will strengthen our knowledge on the role of UMGs and long tail genes more broadly.”*** |

| Reviewer  Comment | There is no justification for the thresholds used by the authors to filter edges in string or humannet or TCGA expression data. |
| --- | --- |
| Author  Response | With the wide increase in the size of biological networks during the last decade, studies started to retain edges that pass relatively high thresholds (compared to 0.4 in a much smaller network in an early study (2008)—PMID 18371930—for instance). Yet, there is no single gold standard threshold and these values vary in the literature. In the manuscript, we retain an equivalent proportion of network edges (~9-10%) compared to ones based on thresholds selected in flagship propagation-based studies that deployed STRING and HumanNet to study cancer genomics (e.g. PMID 24037242).  Similarly, there is no single FPKM cutoff used for expression-based filtering, but these values tend to be moderate to high (i.e. >10) to account for noise and possible alignment errors. We used RNASeqDB data which has been processed to account for batch effects in TCGA, and selected a threshold of FPKM > 15. The same threshold was used in other cancer genomics studies (e.g. PMIDs 26270481 and 22980976). |

| Reviewer  Comment | In 4.7 - a better statistical test here would be Wilcoxon rank sum test which will avoid the need of selecting a rank threshold. |
| --- | --- |
| Author  Response | We thank the reviewer for this insight. We have applied the Wilcoxon rank sum test (i.e. Mann Whitney U test) and updated the manuscript and Supplementary Table 2 accordingly. Results are statistically significant (*p* < 10^-5^) across all PPI-cancer type combinations. |
| Excerpt(s) from Updated Manuscript | **[2.1, paragraph 1] *“[…] Next, we perform the Mann Whitney U test to assess the significance of propagation-based rankings by measuring the enrichment of known functionally important COSMIC genes towards higher ranks in post-propagation lists. Results demonstrate high statistical significance across all studied cohorts (p < 10-5, Supplementary Table 2) demonstrating the validity of the method to identify genes with functional importance.”***  **[4.7, paragraph 1] “*To assess the validity of ranking after propagation, we tested if known COSMIC genes [55] are ranked significantly higher than other genes using the one-sided Mann Whitney U statistical test (also known as one-sided Wilcoxon Rank Sum test). Results show a strong enrichment of COSMIC genes towards highly ranked genes for all PPI network-cancer type combinations (p < 10-5, Supplementary Table 2).”*** |

### Reviewer #2

| Reviewer  Comment | The study by Hussein Mohsen and colleagues uses network analysis in the TCGA dataset to nominate new cancer genes called UMGs (for Upward Mobility Genes). They use the technique of network propagation on gene interaction networks and prioritize genes whose mutation-based ranking becomes stronger once their network context is taken into account through this propagation. They validate their predictions using existing shRNA and CRISPR screens and also present new in vitro validation data but the latter part is underdeveloped. Overall the study is well executed and written and the network-driven approach is interesting as a build-on to existing network propagation method. |
| --- | --- |
| Author  Response | We thank the reviewer for these comments on our study. |

| Reviewer  Comment | 1. Is the network topology of UMGs contributing to their nomination in the cancer-type specific interaction networks? For example, are they hubs in the network or do they have few interaction partners? Are they on frequently used paths in the network? |
| --- | --- |
| Author  Response | We thank the reviewer for this comment. Reported UMGs tend to be well-connected in the network, which provides them with enough upward mobility to be pinpointed by our detection approach. Yet, they do not tend to have a considerably different connectivity from that of non-UMG nodes, except for a small subset of non-UMG hub nodes such as those that correspond to very well-studied cancer drivers. This is evident in Figure 5 (and Supplementary Figures 3-18), where each node’s border thickness corresponds to degree connectivity. Most UMGs (white nodes) have a comparable border thickness to that of most drivers (red nodes), except for *TP53*, *PIK3CA, and BRCA1*, which expectedly stand out with a very thick border. We have updated the manuscript to reflect this observation. Further, a noticeable subset of each cancer type’s UMGs has no edges with known drivers. These UMGs are already placed on the top right of the corresponding figures. |
| Excerpt(s) from Updated Manuscript | **[2.5, paragraph 3] “*We also point out the well-connectedness of many UMGs, which in part allows them to have enough upward mobility to be detected by our approach. Yet, UMGs tend to have a considerably smaller number of neighbors compared to very well-studied drivers such as TP53, PIK3CA, and BRCA1.”*** |

| Reviewer  Comment | 2. How would the method perform on randomly permuted data, for example if mutation counts of genes were to be shuffled around in the networks ? This would help evaluate the false positive rates of such network-informed cancer gene discovery. |
| --- | --- |
| Author  Response | We thank the reviewer for this comment. We partitioned in section 2.2 the 230 UMGs based on their frequency as a UMG across cancer types: 76 (33%) are specific to one cancer type, 116 (50.4%) to 2-9 types, and only 38 (16.5%) to 10 or more types. We expect cancer type-specific UMGs to be with highest confidence among reported genes, while the ones in 10 or more types with a likelihood to include some FPs. The inspection of exploratory permutation results seems to align with these expectations.  We first generated upward mobility lists for 45 permutations per PPI per cancer type (i.e. 45 x 2 x 17 = 1,530 experiments). Within each cancer type, we the identified the list of most frequent *k* UMGs based on permutations, where *k* is the equivalent number of UMGs reported in the manuscript. We then identified the frequency of each actual UMG (i.e. based on TCGA data) in those lists. The majority of UMGs never appeared in these permutation-based lists. Around 30% (73/230) appeared in one or more lists, with this percentage dropping to 25% for ≥ 2 lists and to 21% in ≥ 3 lists. More than 92% of the cancer-specific UMGs (70/76 UMGs in Figure 2, left side) are among the ones that never appeared in permutation-based lists, confirming the high confidence originally associated with them. Further, the majority of the 21% that appeared in ≥ 3 lists (37/48) are among the UMGs reported for a considerable number of cancer types in the manuscript (Figure 2, top right — clockwise from *PPP1CC* to *STAT5A*). However, even these UMGs often appeared in a smaller number of permutation-based lists compared to the cancer types they were reported for. For example, *BIRC5* was identified as a UMG for 6 cancer types while it only appeared in the permutation-based list of one cancer type, suggesting a moderate to high confidence. Another example is *CDH1*, reported as a UMG for 8 types and appearing in 3 permutation-based lists only.  In sum, most UMGs seem to demonstrate high confidence, with the strongest values likely associated with cancer type-specific UMGs. The level of confidence increases in a roughly clockwise direction according to the arrangement of the circus plot in Figure 2. We have updated the manuscript to reflect this observation. |
| Excerpt(s) from Updated Manuscript | **[3, paragraph 3] *“In their current arrangement in the circos plot of Figure 2, we posit that the confidence associated with UMGs increases in a roughly clockwise direction, with the highest confidence to be associated with cancer type-specific genes. The incorporation of additional functional genomics data (e.g. noncoding mutations and methylation data), paralleled with additional studies and an increase in the number of reported PPIs, will likely strengthen our knowledge on the role of UMGs and long tail genes more broadly.”*** |

| Reviewer  Comment | 3. The authors use computationally predicted interaction networks STRING and HumanNet2. While these networks provide a larger search space, their computational origin makes them less reliable and the edges in the network may be difficult to interpret. How robust are their findings to those effects - can they recover the cancer genes if they focused on experimentally defined protein-protein interactions instead? |
| --- | --- |
| Author  Response | We thank the reviewer for this comment. We conducted the requested experiments which also recovered the cancer genes with high significance (*p* < 10^-5^) in the reduced networks. We include the results below following our rationale behind selecting information channels.  The number of reported physical interactions has certainly increased to considerable levels during the last 10 years. In parallel, functional links do represent a valuable source to networks, and in many cases the majority of edges (as with HumanNetv2). To reduce FP rate in our analyses, we implemented a series of stringent filtering steps: edge filtering, expression-based filtering of nodes, and the selection of nodes in largest connected component of the resulting network. We also adopted in the manuscript a common rhetoric in the literature that broadly defines protein-protein interactions to encapsulate both physical interactions and functional associations, as these information channels are often used together (e.g. PMIDs 30476243, 24037242, and 34016094). We have updated the manuscript to clarify this point raised by the reviewer.  Per the reviewer’s request, we have also assessed the ability to recover cancer genes after removing functional edges from both STRING and HumanNetv2 in BRCA, ESCA, and LUAD as examples with well-sized cohorts. For HumanNetv2, we used HumanNetv2-PI which excludes functional associations from the FN version of the network. The drop in high quality edges dramatically amounted to 57.4% (from 37.2 to 15.8K) and that of nodes to 18.5% (from 11.4 to 8.1K). For STRINGv11, we similarly removed functional information channels corresponding to co-expression, fusion, neighborhood, co-occurrence, and text mining. We then recalculated edge confidence scores according to the Bayesian correction approach used in STRING (PMID 15608232). The total number of high quality undirected edges decreased after removing these channels by only 19.1% (from 419.7 to 339.4K), and that of nodes by 14.7% (from 17.1 to 14.6K).  Similar to the results obtained on complete networks, we were able to recover cancer genes (one-sided Mann Whitney U test, *p* < 10^-5^) across both reduced version of STRING and HumanNetv2 and all 3 targeted cancer types. |
| Excerpt(s) from Updated Manuscript | **[Abstract, paragraph 1] *“The study of cancer-relevant genes often requires integrative approaches pooling together multiple types of biological data. Network propagation methods have demonstrated high efficacy in achieving this integration.”***  **[4.3, paragraph 1] *“We adopt the broad definition of protein-protein interactions that encompasses direct physical interactions alongside indirect functional ones derived from co-expression, gene fusion, text mining, co-essentiality, and pathway membership datasets among others.”***  **[3, paragraph 3] *“The incorporation of additional functional genomics data (e.g. noncoding mutations and methylation data), paralleled with additional studies and an increase in the number of reported PPIs, will likely strengthen our knowledge on the role of UMGs and long tail genes more broadly.”*** |

| Reviewer  Comment | 4. The study focuses on protein-coding mutations, but is it extendable to non-coding mutations? A recent study of the PCAWG used network and pathway-based approaches to nominate cancer genes in the long tail, including network propagation techniques. |
| --- | --- |
| Author  Response | Yes. Our UMG detection method is extendable to PCAWG and numerous other sources of functional genomics data at the cohort or sample level. While its design was inspired by the long tail gene prioritization problem in cancer, the method could in fact be applied to other problems in medicine and beyond to detect ‘silent players’ in a given network by providing a set of initial node scores. We hope our method and results could be inspiring for future research in this direction. |
| Excerpt(s) from Updated Manuscript | **[3, paragraph 3] *“The incorporation of additional functional genomics data (e.g. noncoding mutations and methylation data), paralleled with the continuous increase in the number of PPIs and functional associations, could also strengthen our knowledge on UMGs’ role and that of long tail genes in general.”*** |

| Reviewer  Comment | 5. The experimental validation data is described only in 10 lines and therefore not as convincing. How were the genes selected - it seems that some were the genes they predicted and others were somehow related genes? What controls were used? Is it expected to see such level of UMGs and others being validation? This part would substantially increase the value of the entire study in the opinion of the reviewer, but its current level of development is too limited to add this value. |
| --- | --- |
| Author  Response | We thank the reviewer for his/her comment. We have almost doubled the size of our *in vitro* experiments in this version and substantially updated the corresponding manuscript sections as described below.  Up to section 2.6, our validation of UMG lists across cancer types is based on the comprehensive set of cell lines provided by the DepMap Project: 990 cell lines for CRISPR-based assays and 712 lines for RNAi-based ones. We updated section 2.4 to ensure it includes these details.  We then performed experimental *in vitro* validation of a specific subset of UMGs that have never been reported to be tested in gene knockdown experiments in the context of any cancer phenotype. We identified these UMGs (n=29) after a manual search of PubMed literature according to the criterion we describe in updated section 4.13. Relatedly, we apologize for the confusion that stemmed from including a small number (n=4) of functionally related non-UMGs in the previous manuscript. For clarity, we excluded these genes and restricted our focus on the 29 UMGs.  We conducted new experiments on lung and prostate cancer cell lines (n=3). This almost doubled the size of experiments previously reported for this section (from 4 to 7 cell lines) and added new primary sites as previous results were restricted to breast cancer lines. We list all cell lines and ensure both negative and positive controls used in these experiments are mentioned in section 4.14 of the updated manuscript.  The new expanded set of results brings the number of validated UMGs from 4 in the previous manuscript up to 18 in the updated one. As members of this UMG subset were never targets of knockdown experiments that study their effects on cancer phenotype earlier, we expected a handful of genes to be validated. A number as high as 18 exceeded our expectations a bit and further highlights the potential importance of identified UMGs. We list these 18 genes in updated section 2.7 and include detailed experimental results in Supplementary Table 23. |
| Excerpt(s) from Updated Manuscript | [**2.4, paragraph 1] *“The CRISPR dataset includes 990 cell lines, and the RNAi dataset includes 712 cell lines.”***  **[2.7, paragraph 1] *“We performed a series of siRNA knockdown experiments in vitro to validate the DepMap results and to confirm the functional importance of selected UMGs. We selected 29 UMGs that have not been reported in the literature to be tested in gene knockdown experiments in the context of any cancer phenotype (Methods). We used 7 cell lines representing 3 types of cancer, namely H460 and HCC1299 from lung, MDAMB231, MDAMB468, BT549, and HCC187 from breast, and DU145 from prostate cancer.”***  **[2.7, paragraph 2] *“Experimental results further underscore the efficacy of UMG detection to uncover functionally important long tail genes. The knockdown of 18 out of these 29 UMGs (62%) significantly decreased cell survival in 1 to 5 cell lines exceeding the threshold of 3 standard deviations with respect to negative control samples (Methods, Supplementary Table 23). We note that several UMGs demonstrated cell line specificity while others had a more widespread effect (affects 5/7 cell lines). These newly cancer-relevant genes have already known functions in regulating immune response (AP2M1, DCTN1, CCT4, DYNC1I2, and DYNC1LI2), kinase binding (DLG3), cell cycle progression (SEC13, ANAPC7, CDC26, PSMC3, PPP1CC), DNA repair (PPIE, RFC5, POLR2E and POLR2L), cell death (VAPA), and mRNA splicing (SF3A2). The list also includes PNPLA2, which encodes for an enzyme associated with transacetylase activity.”***  **[4.13, paragraph 1] *“We manually cross referenced each UMG with PubMed publications to detect which ones have been earlier reported to play a role in cancer based on functional experiments. We based results on an extensive search using the gene name AND “cancer” as keywords in PubMed. If any gene was the target of a previous functional assay, i.e. was deliberately overexpressed, suppressed, or mutated, and resulted an in vitro change in the proliferation or survival of cancer cell lines, it was annotated as functionally validated. Otherwise, the genes is considered not validated. Fully annotated UMG lists for each of the 17 cancer types are provided in Supplementary Tables 5-21, while the list of publications associated with functionally validated UMGs is provided in Supplementary Table 22.”***  **[4.14, paragraph 1] *“Cell lines from breast (MDAMB231, MDAMB468, BT549, HCC187), lung (H460, HCC1299), and prostate cancers (DU145) were cultured in RPMI medium supplemented with 10% HI-FBS and penicillin/streptomycin (1:100).”***  **[4.14, paragraph 1] *“Cells were then seeded at plating densities optimized during assay development (MDAMB468, HCC1187, and BT549 seeded at 4000 cells per well; MDAMB231 and H460 seeded at 1000 cells per well; DU145 and HCC1299 seeded at 500 cells per well) and incubated at 37C. After 72 hours, CellTiter-Glo (Promega) was used to monitor viability. Each screening plate contained 16 replicates of negative siRNA controls (either siGENOME Smart Pool non-targeting control #1, #2, or #4, Dharmacon) and positive siRNAs controls (siGENOME Smart Pool Human PLK1 or KIF11, Dharmacon).”*** |

| Reviewer  Comment | 6. The gene set enrichment analysis is performed using the DAVID software that employs databases that are more than five years old. The authors are likely to miss out on a substantial fraction of functional annotations of their UMGs. |
| --- | --- |
| Author  Response | We thank the reviewer for this comment. We primarily chose DAVID because it pools results from multiple sources into a unified format that is among the very few formats compatibile with the EnrichmentMapv3.0 plugin in Cytoscape. This allowed us to visualize enrichment results in a network form (Figure 3, panels b and c). As our analyses have an exploratory nature to prioritize less studied long tail genes, we limited enrichment results to well-studied biological functionalities (related to cancer or essential processes) currently available in DAVID from highly reliable KEGG and GO.  While DAVID remains one of the very popular annotation databases—with a number of citations that peaked at 6,000+ in 2020 alone—we are optimistic that additional analyses based on other resources will be available in the near future and will broaden our understanding of long tail genes’ significance. |

**Second round of review**

**Reviewer 1**

I like this paper, but I think it would benefit from additional evidence for the advantage of UMGs over standard propagation.

While the authors nicely compared to PPR - the exact details of the latter may differ from the authors' own propagation algorithm and the method may suffer from tendency to give high-scores to hubs. This plus additional filters the authors employ (depmap score, removal of driver genes, inclusion of initial low-scoring genes) may explain the difference (albeit small) in performance between the methods. Since the authors produce final scores for genes following propagation they can directly compare their "plain" UMG list (i.e., no filters and after removal of initial low-scoring genes) to a same-size RFS list and report the results a la Figure 4. For the latter RFS list I would be interested to see two versions: (i) as is; and (ii) normalized for the degree-bias, so that the score of a gene after propagation is divided by its score when alpha=1 (i.e., the propagation accounts for the network only and ignores the initial scores).

**Reviewer 2**

The authors have addressed my comments and improved their manuscript. One comment remains: the DAVID resource should be not used (comment 6). The continued use of outdated functional annotations is harmful since it can miss the majority of associations in enrichment analysis (https://www.nature.com/articles/nmeth.3963).

**Authors Response**

**Point-by-point responses to the reviewers’ comments:**

### Reviewer #1

| Reviewer  Comment | The authors have addressed most of my comments in a satisfactory manner. The remaining major comment is described below in the comments for authors.  I like this paper, but I think it would benefit from additional evidence for the advantage of UMGs over standard propagation.  While the authors nicely compared to PPR - the exact details of the latter may differ from the authors' own propagation algorithm and the method may suffer from tendency to give high-scores to hubs. This plus additional filters the authors employ (depmap score, removal of driver genes, inclusion of initial low-scoring genes) may explain the difference (albeit small) in performance between the methods. Since the authors produce final scores for genes following propagation they can directly compare their "plain" UMG list (i.e., no filters and after removal of initial low-scoring genes) to a same-size RFS list and report the results a la Figure 4. For the latter RFS list I would be interested to see two versions: (i) as is; and (ii) normalized for the degree-bias, so that the score of a gene after propagation is divided by its score when alpha=1 (i.e., the propagation accounts for the network only and ignores the initial scores). |
| --- | --- |
| Author  Response | We thank the reviewer for this comment, and we are glad that s/he likes our paper. We have conducted more method comparisons, all of which confirmed the outperformance of UMG gene sets over to those identified by other methods.  We have previously included the top genes from as-is RFS lists yet under the name of PPR. As many variants of PPR and Random Walk with Restart emerged during the last two decades, both terms have been colloquially used to refer to the class of methods that share the same spirit and vary slightly on the technical level. To ensure clarity, we renamed the propagation method based on which as-is RFS lists are generated to Zhou *et al* throughout the manuscript. Relatedly, we have also implemented the edge-normalized version the reviewer suggested and incorporated its results in Figure 4 under “Zhou et al-EN.” UMGs performed better by far across cancer types.  In relation to gene filtering, we compared UMGs both before the final DepMap filtering step and after including drivers to all other methods’ lists. Results are very similar to the ones in Figure 3, and we included them in a new supplementary figure. As for initial low scoring genes, we recognize where the reviewer is coming from, yet these genes are by definition integral to the long tail of the mutational distribution, are not usually excluded by other methods, and include UMGs that are highly potential drug targets. We still conducted inspection experiments without these genes on the three cancer types we used in the previous review cycle, namely BRCA, ESCA, and LUAD, and these experiments similarly confirmed UMGs’ considerable outperformance over its counterparts with little to no impact on results across the board.  The manuscript, Figure 4, and Supplementary Figure 3 have been updated/added to reflect these changes accordingly. |
| Excerpt(s) from Updated Manuscript | **[2.4, paragraph 3] *“To have a better understanding of the specifications of UMGs, we compared their impact on the survival of cancer cell lines to that of non-driver genes selected by five other methods. Three of these methods are propagation-based and include FDRNet [44], Hierarchical HotNet (HHotNet) [22]—in 3 different settings, and Zhou et al.’s propagation algorithm that resembles random walk with restart—in its original and edge-normalized settings [45].”***  **[2.4, paragraph 4] “*Similar results were also obtained for these comparisons before the optional DepMap filtering step that only removed 4-13% of UMGs (Supplementary Figure 1). As FDRNet, HHotNet, Zhou et al., nCOP and MutSig do not solely focus on long tail genes and gene sets generated by these methods include known cancer drivers, we performed the same comparisons after including known cancer-specific drivers from all gene lists, which also produced similar results (Supplementary Figure 2). Concurrently including both subsets of UMGs (pre-DepMap filtering and drivers) produced similar results across cancer types as well (Supplementary Figure 3).”***  **[4.10, paragraph 1] *“For Zhou et al.’s propagation method, we select the top k genes identified post-propagation, where k is the equivalent number of UMGs for each cancer type across networks. In its edge normalized setting, we divide each gene’s post-propagation score by the same score when propagation α = 1 (i.e. ignoring initial scores) before selecting top genes.”*** |

### Reviewer #2

| Reviewer  Comment | The authors have addressed my comments and improved their manuscript. One comment remains: the DAVID resource should be not used (comment 6). The continued use of outdated functional annotations is harmful since it can miss the majority of associations in enrichment analysis (<https://www.nature.com/articles/nmeth.3963>). |
| --- | --- |
| Author  Response | We thank the reviewer for this comment and for the eye-opening reference s/he shared. We have re-generated enrichment results based on g:Profiler with all information channels updated in 2021.  The manuscript and Figure 3 have been updated accordingly. |
| Excerpt(s) from Updated Manuscript | **[4.11, paragraph 1] “*Enrichment analysis to identify pathways, GO molecular functions, and GO biological processes is performed on g:Profiler [56]. Enrichment results with Benjamini p-adjusted < 0.05 are selected for analysis. Network visualization is executed using EnrichmentMap v3.0 on Cytoscape v3.8.2 [57 [...]”*** |

**Third round of review**

**Reviewer 2**

The authors have addressed my remaining comment and I am happy to recommend publication. I was asked by the editor to review the comments of the other reviewer as well. Apparently those comments are also resolved and I have no further concerns.
